# Impact of Preconception Micronutrient Supplementation on Anemia and Iron Status during Pregnancy and Postpartum: A Randomized Controlled Trial in Rural Vietnam

**DOI:** 10.1371/journal.pone.0167416

**Published:** 2016-12-05

**Authors:** Phuong H. Nguyen, Melissa Young, Ines Gonzalez-Casanova, Hoa Q. Pham, Hieu Nguyen, Truong V. Truong, Son V. Nguyen, Kimberly B. Harding, Gregory A. Reinhart, Reynaldo Martorell, Usha Ramakrishnan

**Affiliations:** 1 Poverty, Health and Nutrition Division, International Food Policy Research Institute, Washington, DC, United States of America; 2 Thai Nguyen University of Pharmacy and Medicine, Thai Nguyen, Vietnam; 3 Hubert Department of Global Health, Rollins School of Public Health, Emory University, Atlanta, GA, United States of America; 4 Micronutrient Initiative, Ottawa, Ontario, Canada; 5 The Mathile Institute for the Advancement of Human Nutrition, Dayton, OH, United States of America; United States of America; Kaohsiung Medical University Hospital, TAIWAN

## Abstract

**Objective:**

Preconception micronutrient interventions may be a promising approach to reduce anemia and iron deficiency during pregnancy, but currently we have limited data to inform policies. We evaluated whether providing additional pre-pregnancy weekly iron-folic acid (IFA) or multiple micronutrient (MM) supplements compared to only folic acid (FA) improves iron status and anemia during pregnancy and early postpartum.

**Methods:**

We conducted a double blind randomized controlled trial in which 5011 Vietnamese women were provided with weekly supplements containing either only 2800 μg FA (control group), IFA (60 mg Fe and 2800 μg FA) or MM (15 micronutrients with similar amounts of IFA). All women who became pregnant (n = 1813) in each of the 3 groups received daily IFA (60 mg Fe and 400 μg FA) through delivery. Hematological indicators were assessed at baseline (pre-pregnancy), during pregnancy, 3 months post-partum, and in cord blood. Adjusted generalized linear models were applied to examine the impact of preconception supplementation on anemia and iron stores, using both intention to treat and per protocol analyses (women consumed supplements ≥ 26 weeks before conception).

**Results:**

At baseline, 20% of women were anemic, but only 14% had low iron stores (ferritin <30 μg/L) and 3% had iron deficiency (ferritin <12 μg/L). The groups were balanced for baseline characteristics. Anemia prevalence increased during pregnancy and post-partum but was similar among intervention groups. In intention to treat analyses, prenatal ferritin was significantly higher among women receiving MM (geometric mean (μg/L) [95% CI]: 93.6 [89.3–98.2]) and IFA (91.9 [87.6–96.3]) compared to control (85.3 [81.5–89.2]). In per protocol analyses, women receiving MM or IFA had higher ferritin 3 months postpartum (MM 118.2 [109.3–127.8]), IFA 117.8 [108.7–127.7] vs control 101.5 [94.0–109.7]) and gave birth to infants with greater iron stores (MM 184.3 [176.1–192.9]), IFA 189.9 [181.6–198.3] vs control 175.1 [167.9–182.6]).

**Conclusion:**

Preconception supplementation with MM or IFA resulted in modest increases in maternal and infant iron stores but did not impact anemia. Further research is needed to characterize the etiology of anemia in this population and identify effective interventions for reducing prenatal anemia.

**Trial Registration:**

ClinicalTrials.Gov NCT01665378

## Introduction

Globally an estimated 32 million pregnant women and 273 million children under five are anemic [1]. In South East Asia, the burden of anemia is particularly high and more than 1 in 4 women suffer from anemia during pregnancy. The causes of anemia are varied but iron deficiency has been identified as the most common and has been linked with increased risk of maternal and perinatal mortality and adverse birth outcomes [2–5]. Iron supplementation during pregnancy is a common strategy to combat anemia. Evidence from controlled trials have shown that this strategy can reduce the incidence of anemia and iron deficiency anemia at term by nearly two-thirds and improve birth outcomes [6–8]. However maternal anemia remains a significant public health problem across the globe. Poor program implementation and utilization have been identified as key limiting factors for the success of these programs. In a review of 2003–2009 Demographic and Health Survey data, less than 25% of women in South/South East Asia received iron supplements for at least 90 days during pregnancy [9]. Often by the time a woman seeks prenatal care, it may be too late to build iron stores with iron and folic acid (IFA) supplements given the high iron demands of pregnancy. Furthermore, women may also miss the critical preconception/early pregnancy period for improving folate status to reduce neural tube defects [10].

There is growing recognition on the importance of adolescent and women’s preconception nutritional status to improve maternal and infant outcomes [11–15]. Women who enter pregnancy with suboptimal iron stores may be at higher risk for poor maternal and infant outcomes [16]. The World Health Organization recommends intermittent IFA supplementation for menstruating women as one of the strategies to reduce anemia and improve iron status among women of reproductive age (WRA), in settings where anemia prevalence in this population is over 20% [17]. This recommendation was informed by evidence from controlled trials which showed that intermittent IFA reduced the risk of anemia by an average of 27%, in addition to improving hemoglobin and ferritin concentrations [18]. Program experience with this policy from several countries including Vietnam, Cambodia, Egypt, India, Laos and Philippines have reported a wide range in average decreases in anemia (9% to 57%) [19]. In addition, providing WRA with weekly IFA pre-pregnancy significantly reduced iron deficiency for the first two trimesters of pregnancy in Vietnam [20] and improved iron status during pregnancy in Cambodia and Philippines [21,22].

In addition to the growing evidence on the potential of providing IFA supplements, preconception multiple micronutrients (MM) may also be an effective strategy for improving pregnancy and birth outcomes [13]. Women living in regions with endemic anemia often suffer from multiple nutrient deficiencies beyond just iron deficiency given the overall poor diet quality and limited access to sufficient nutrient rich foods. The 2015 Cochrane review on MM supplementation during pregnancy included 17 trials (involving 137,791 women) primarily in low and middle-income countries and concluded that compared to IFA or folic acid (FA), MM supplementation significantly decreased LBW (12%), small-for-gestational age (SGA, 10%) and stillbirth (9%) [23]. The use of daily MM supplements during pregnancy has also been associated with a significant increase of 52.6 g in birth weight compared to IFA supplementation alone [13]. In addition, observational studies suggest that consumption of MM supplements before or during the time of conception is associated with a reduced risk of fetal growth restriction, preterm birth and low birth weight [13]. However while there is compelling evidence for MM supplementation during pregnancy, the overall quality of the preconception evidence is low with little or no information from well-designed randomized controlled trials (RCT).

Thus, there remain several gaps in our understanding of the impact of pre-pregnancy supplementation and there is need for evidence based interventions in the preconception period [24]. In order to address these gaps, we conducted a large randomized controlled trial (PRECONCEPT study) evaluating the effects of preconceptional micronutrient interventions on maternal and child health outcomes in rural Vietnam [25]. We have previously reported the effect of the intervention on birth outcomes [26]. The objective of this paper is to examine the impact providing additional pre-pregnancy weekly iron-folic acid (IFA) or multiple micronutrients (MM- 15 vitamins and minerals; including IFA) compared to only folic acid (FA) on improving iron status and anemia during pregnancy and early postpartum in mothers and at birth in their offspring.

## Subjects and Methods

### Study design, setting and participants

The study design, trial profile and characteristics of the study sample have been described in detail elsewhere [25]. Briefly, we conducted a randomized, double-blind controlled trial in Thai Nguyen, a mountainous province in the north of Vietnam. A total of 5011 currently married women aged 18–40 years, who were planning on becoming pregnant in the next year, were recruited from 20 communes selected from four districts in Thai Nguyen province. Exclusion criteria included being pregnant at the time of recruitment, consuming IFA or MM supplements in the previous 2 months, being severely anemic (Hb < 7 g/L), and having history of high risk pregnancy or chronic hematological diseases. Women with severe anemia were referred to the local health clinic for treatment.

All eligible women were randomly assigned to one of three groups for weekly pre-pregnancy supplementation: 1) folic acid (FA, 2800 μg–control), 2) iron and folic acid (IFA, 2800 μg FA+ 60 mg iron) or 3) multiple micronutrients (MM). The composition of the MM supplement was based on the UNIMMAP international multiple micronutrient preparation [27] used in daily prenatal trials with a few modifications, namely the same amounts of iron and folic acid as the weekly IFA supplement and a higher amount of Vitamin D (600 IU) to be consistent with the recent Institute of Medicine recommendations [28]. The micronutrient composition of the supplements is shown in Table 1. Before the start of the supplementation protocol, all participants were dewormed with albendazole (400 mg) to treat parasitic infections that were prevalent in the study settings [29]. Once women became pregnant they stopped weekly preconception supplementation and started receiving prenatal supplements containing 60 mg of iron and 400 ug of FA to be taken daily through delivery as recommended by World Health Organization WHO [30]. The supplement preparations (MM, IFA, and FA) were physically indistinguishable. The tablet containers were labeled by the manufacturer with letter and color codes that were not disclosed to the researchers until the data collection as per the original study design was complete (when the youngest child in the cohort turned 3 mo). All investigators, study personnel and participants were blinded to group assignment.

**Table 1:** The composition of iron folate and multiple micronutrient supplements.

Ingredient	Pre-pregnancy (weekly)			Vietnamese RDA for non-pregnant women^1^ [31]
	FA	IFA	MM	
Vitamin A (μg)			800	500
Vitamin D (IU)			600	15
Vitamin E (mg)			10	12
Vitamin C (mg)			70	70
Thiamine (mg)			1.4	1.1
Riboflavin (mg)			1.4	1.1
Niacin (mg)			18	14
Vitamin B_6_ (mg)			1.9	1.3
Vitamin B_12_ (μg)			2.6	2.4
Folic acid (μg)	2800	2800	2800	400
Iron (ferrous sulfate) (mg)		60	60	29.4^**1**^
Zinc (sulfate) (mg)			15	9.8^**1**^
Copper (mg)			2	900
Selenium (μg)			65	55
Iodine (μg)			150	150

FA—folic acid, IFA—iron and folic acid, MM—Multiple micronutrient, RDA—Recommended dietary allowance.

^1^RDA for Vietnamese women are similar to US RDA, except for higher iron (18 mg/day) and zinc (8 mg/day) due to assumptions on lower dietary bioavailability).

The study was approved by the Ethical Committee of Institute of Social and Medicine Studies in Vietnam (May 31, 2011) and Emory University's Institutional Review Board, Atlanta, Georgia, USA (July 26, 2011). Written informed consent was obtained from all study participants. Baseline recruitment was from October 15, 2011 to April 8, 2012 and all follow up was completed in September 13, 2014. The study was registered at ClinicalTrials.Gov as NCT01665378. Although the registration occurred slightly after the study began, no changes were made to the planned design between the baseline data collection and trial registration.

### Compliance

Village health workers (VHWs) visited women every two weeks to deliver supplements, monitor compliance, and record any symptoms or side effects women may have. The VHW directly observed the consumption of one tablet during their visit, and called or used text messages the weeks in between her visits to remind the participants to take the subsequent tablet. Compliance was based on a count of empty capsule packets stored in the women’s homes. Women were encouraged to consume supplements on an empty stomach at the same time every week to enhance compliance, absorption, and minimize side-effects. VHWs also asked women about their menses since the last visit and referred women to Commune Health Center for pregnancy test and prenatal care if women reported that their last menstrual period had occurred >5 weeks.

### Outcomes

Hemoglobin concentrations were measured from capillary blood samples (by finger prick) at baseline (preconception), each trimester during pregnancy, and 3 months postpartum for both women and their children using a portable field B-Hemoglobin Analyzer [32]. Maternal anemia was defined as a Hb value <12 g/dL for baseline and postpartum, and <11 g/dL during pregnancy [33]. Maternal high Hb concentrations at any time during pregnancy was defined as Hb >13 g/dL) [8]. For children, anemia at 3 months was defined as Hb < 11 g/dL [33].

Maternal iron status was assessed at baseline, during the first prenatal visit and 3 months post-partum. Venous blood samples (5 ml) were collected using heparin-treated vacutainers after an overnight fasting. Child iron status was assessed at delivery using cord blood samples (5 ml). All blood samples were obtained by trained staff at the Commune Health Center or District Hospital, stored in an icebox and transported within 4 h to TUMP’s Hematology Department where they were centrifuged at 1500g. Aliquots of plasma were stored in bar-coded vials at −70°C until further analysis. Plasma ferritin and transferrin receptor (TFR) were measured using the sandwich ELISA technique [34] at the Erhardt Laboratory in Germany. The intra-assay coefficient of variation was <3.0%. Low iron stores was defined as plasma ferritin <30 μg/L and iron deficiency as plasma ferritin <12 μg/L [35,36] while high iron stores was defined as ferritin >150 μg/L [8]. The cutoff for TFR from the commercial kit to define iron deficiency was >8.3 mg/L.

### Covariates

Extensive data on socio-demographic characteristics including socioeconomic status, age at marriage and enrollment, ethnicity, education and occupation were obtained for all women at baseline [25]. Detailed obstetric and health history and anthropometric measurements were also obtained at baseline and the first prenatal visit. Dietary intakes were assessed at baseline using a semi-quantitative food frequency questionnaire that was developed and validated for Vietnamese population [37] and administered by trained field workers.

The markers of inflammation, serum C- reactive protein (CRP) and alpha-1-acid glycoprotein (AGP) were measured using the sandwich ELISA technique [34]. CRP and AGP, were considered to be elevated at >5 mg/l and >1 g/l, respectively [38].

### Statistical analysis

Data were checked for normality using the Kolmogorov-Smirnov test of normality. Log transformation was used to normalize the distribution of plasma ferritin, and the geometric means and 95% CI were reported. Means (± SD) and proportions for selected baseline variables were compared between treatment groups using Analysis of Variance (ANOVA) and chi-square test, respectively, to determine if the groups were similar. Adjusted generalized linear models were used to examine the impact of preconception supplementation on anemia and iron stores. Data were first analyzed using intention to treat approach for all the outcomes of interest, followed by per protocol comparisons that took into account a pre-specified minimum duration of intervention, namely at least 26 weeks before conception. We also adjusted for gestational age (because iron markers are measured at different time point of pregnancy), duration of supplements (to account for varying time from enrollment to conception in different groups) and inflammation indicators (to account for the effects of infection on iron stores) in all models. Finally, we investigated whether the effects of supplementation varied by baseline anemia, iron and nutrition status (BMI <18.5 vs. BMI ≥18.5). Statistical analysis was carried out using Stata (version 13) and SAS (SAS for Windows, version 9.3, SAS Institute).

## Results

**Fig 1:**
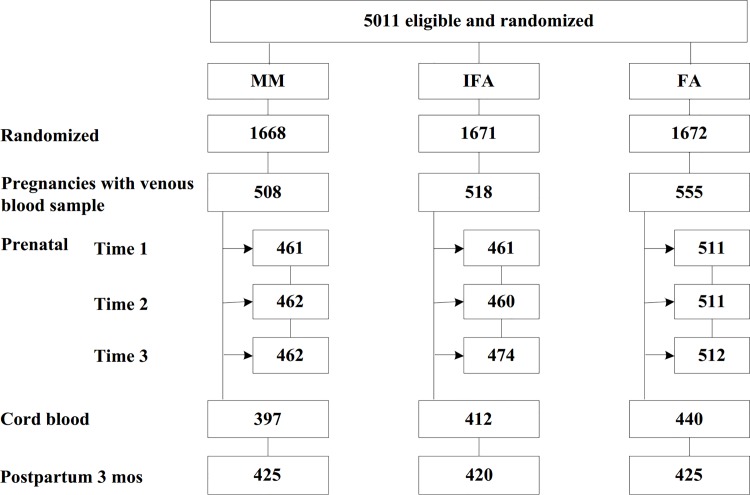
Flow diagram of participant progress throughout the study. FA- Folic Acid, IFA- Iron and Folic Acid, MM- Multiple Micronutrient.

Among 5,011 women randomized for inclusion into the study, 1,813 women became pregnant and contributed 1,599 birth outcomes. Intent to treat analyses was done for 1,581 women with venous blood samples at the first prenatal visit, 1,249 women with cord blood samples and 1,291 women with venous blood samples at 3 month postpartum (Fig 1).

Selected baseline characteristics including age, education, occupation, ethnicity, dietary intakes and anthropometric measurements were not significantly different across the three treatment groups (Table 2). Women were on average 26 years old and married at age 22; almost half belonged to an ethnic minority, around 80% had one child, approximately 30% had low body mass index (BMI <18.5 kg/m^2^), approximately 80% worked as farmers, and over a third graduated from high school. There were no differences in baseline characteristics by treatment group at any time point (enrollment, pregnancy cohort, women with known birth outcomes, women with measurements at 3 mo postpartum and women with cord blood measurements (data not shown).

**Table 2:** Maternal characteristics at baseline by intervention group^1^.

Variable	MM	IFA	FA
	(n = 508)	(n = 518)	(n = 555)
**Age at randomization, y**	26.20 ± 4.70	26.02 ± 4.20	25.76 ± 4.24
**Age at first marriage, y**	21.74 ± 3.27	21.83 ± 3.30	21.85 ± 3.30
**Ethnic minority, %**	52.66	48.64	47.20
**Education Level, %**			
Primary school	10.63	12.98	13.56
Secondary school	26.97	25.58	23.69
High school	53.35	53.68	55.70
College or higher	9.06	7.75	7.05
**Work as farmers, %**	81.50	78.68	77.76
**Having at least one child, %**	79.92	81.59	78.48
**Anthropometric measurements**			
Height, cm	152.8 ± 5.00	152.6 ± 5.05	152.9 ± 5.20
Weight, kg	46.17 ± 5.73	45.46 ± 5.18	45.82 ± 5.44
BMI, kg/m^2^	19.77 ± 2.06	19.52 ± 1.91	19.60 ± 2.10
BMI<18.5, %	29.22	30.74	31.88
**Macronutrient intake/day**			
Total energy, kcal	2241 ± 786	2245 ± 751	2202 ± 716
Carbohydrate (g)	362.54 ± 107.85	359.38 ± 113.11	357.75 ± 110.14
Fat (g)	50.30 ± 31.43	51.67 ± 25.89	49.13 ± 25.76
Protein (g)	84.44 ± 36.19	85.39 ± 33.81	82.03 ± 31.41
**Micronutrient intake/ day**			
Iron, mg	17.36 ± 8.26	17.74 ± 7.64	17.26 ± 8.13
Zinc, mg	10.61 ± 3.62	10.62 ± 3.44	10.40 ± 3.58
Vitamin C, mg	236 ± 164	246 ± 145	250 ± 201
Folate, mg	343 ± 211	356 ± 192	349 ± 196
Vitamin B12, μg	2.15 ±2.33	2.01 ± 1.78	1.97 ± 1.85
Vitamin A, μg	925.59 ± 730.47	955.27 ± 647.19	968.57 ± 813.34
**% < EAR [39]**			
Iron, %	25.79	23.17	24.68
Zinc, %	14.96	16.41	16.76
Vitamin C, %	5.12	3.67	6.31
Folate, %	55.51	50.97	51.17
Vitamin B12, %	62.40	62.93	64.32
Vitamin A, %	29.33	26.64	24.32
**Hematological indicators**			
Hemoglobin, g/dL	12.91 ± 1.38	12.91 ± 1.38	13.04 ± 1.26
Anemia, %	21.43	20.00	17.18
Plasma TFR, mg/L	4.90 ± 1.82	4.74 ± 1.48	4.67 ± 1.56
Plasma TFR >8.3, %	3.97	3.12	1.81
Plasma Ferritin^2^, μg/L	66.31 (61.92, 71.01)	64.07 (60.09, 68.32)	66.89 (61.98, 70.04)
Low iron store (ferritin <30 μg/L), %	13.41	14.04	11.21
Iron deficiency (ferritin <12 μg/L), %	3.55	2.92	2.71
Iron deficiency anemia, %	1.59	1.95	1.08
Plasma RBP, μmol/L	1.61 ± 0.42	1.62 ± 0.44	1.62 ± 0.40
Acute inflammation (CRP >5 mg/L), %	3.16	2.53	2.89
Chronic inflammation (AGP >1 g/L), %	4.93	5.65	4.16

^1^ Sample of women with venous blood samples at the 1^st^ prenatal visit. Values are mean ± SD or percentages. No significant differences among all baseline variables using ANOVA test for comparison of means and goodness of fit test for comparison of proportions, P >0.05.

^2^ Geometric mean (95% Confidence Interval)

AGP- alpha(1)-acid glycoprotein, BMI- Body Mass Index, CRP- C-reactive Protein, EAR: Estimated Adequate Requirement for US (no Vietnamese specific cut points for EAR), FA- Folic Acid, Hb- Hemoglobin, IFA- Iron and Folic Acid, MM- Multiple Micronutrient, RBP- Retinol Binding Protein, TFR- Transferrin Receptor.

Approximately 20% had anemia at baseline, but only 14% had low iron stores and 3% had iron deficiency. Plasma CRP and AGP concentrations were also low (less than 3% of CRP values were >5 mg/L and less than 6% AGP values >1g/L) (Table 2). There were no significant differences by treatment group at baseline for the various biomarkers of iron status, hemoglobin concentrations, inflammation, gestational age or time between preconception enrollment and first prenatal visit.

The average duration of consumption of the weekly preconcept supplements and daily prenatal IFA supplements was 56 ± 28 weeks and 28 ± 8 weeks, respectively. The duration of preconception supplementation was 2 weeks shorter in the MM group. Compliance was relatively high; 78% of the women consumed more than 80% of the preconception supplements and there were no differences in compliance by intervention group both before conception and during pregnancy.

In the intent to treat analysis, the ferritin concentrations at first prenatal visit was significantly higher in the MM (geometric mean (μg/L) [95% CI]: 93.6 [89.3–98.2]) and IFA (91.9 [87.6–96.3]) compared to control (85.3 [81.5–89.2]) (*P* = 0.045) (Table 3). TFR was also higher in the MM group compared to the IFA and FA groups at prenatal (*P* = 0.006) and postnatal visits (*P* = 0.024). No significant differences were observed for hemoglobin or anemia.

**Table 3:** Hematological indicators from preconception to postpartum by intervention group (intention to treat analyses) ^1^.

Variable	MM	IFA	FA
	(n = 508)	(n = 518)	(n = 555)
**Hemoglobin (g/dL)**			
Prenatal at 1^st^ trimester (<14 wks)	12.14 ± 1.40	11.93 ± 1.25	11.96 ± 1.35
Prenatal at 2^nd^ trimester (14–27.9 wks)	11.22 ± 1.31	11.20 ± 1.25	11.29 ± 1.18
Prenatal at 3^rd^ trimester (≥28 wks)	11.46 ± 1.31	11.48 ± 1.30	11.53 ± 1.31
Postpartum (3 mo)- Mothers	12.33 ± 1.27	12.34 ± 1.24	12.35 ± 1.28
Postpartum (3 mo)- Children	10.65 ± 1.36	10.64 ± 1.25	10.58 ± 1.33
**Anemia (%)**^**2**^			
Prenatal at 1^st^ trimester (<14 wks)	17.95	21.74	17.87
Prenatal at 2^nd^ trimester (14–27.9 wks)	37.64	40.75	39.67
Prenatal at 3^rd^ trimester (≥28 wks)	33.79	33.20	31.62
Postpartum (3 mo)- Mothers	38.55	39.33	36.57
Postpartum (3 mo)- Children	42.12	41.35	43.28
**Plasma Ferritin**^**3**^ **(μg/L)**			
Prenatal	93.62^a^ (89.27, 98.18)	91.85^a^ (87.59, 96.33)	85.30^b^ (81.54, 89.23)
Postpartum (3 mo)	114.11 (108.76, 119.73)	112.70 (107.35, 118.32)	109.68 (104.53, 115.09)
Cord blood	178.88 (172.87, 185.10)	184.19 (178.05, 190.54)	175.12 (169.45, 180.98)
**Plasma transferrin receptor (μg/L)**			
Prenatal	3.64 ± 1.24^a^	3.44 ± 1.24^b^	3.44 ± 1.22^b^
Postpartum (3 mo)	5.03 ± 1.34^a^	4.85 ± 1.34^b^	4.83 ± 1.34^b^
Cord blood	7.67 ± 2.90	7.30 ± 2.90	7.50 ± 2.88

^1^Values are mean ± SD for hemoglobin, and transferrin receptor, percentages for anemia and geometric mean and 95% CI for ferritin. Difference among treatment groups were tested using generalized linear regression, adjusting for gestational age, duration of supplements and inflammation. Significant differences (p<0.05) between groups are denoted by different letter superscripts

^2^Non-pregnant women: Hb<12 g/dL; pregnant women: Hb<11 g/dL; children at 3 months: Hb<11 g/dL

^3^ Geometric mean (95% Confidence Interval). FA- Folic Acid, IFA- Iron and Folic Acid, MM- Multiple Micronutrient

Among women who consumed preconception supplements for at least 26 weeks (per protocol analysis, n = 824), plasma ferritin was not different between treatment groups during pregnancy, but it was significantly higher in cord blood (*P* = 0.039) and for mothers at 3 mo postpartum (*P* = 0.031) in the MM or IFA groups compared to FA group (Table 4). Women receiving MM or IFA had higher ferritin 3 months postpartum (MM 118.2 [109.3–127.8]), IFA 117.8 [108.7–127.7] vs control 101.5 [94.0–109.7]) and gave birth to infants with greater iron stores (MM 184.3 [176.1–192.9]), IFA 189.9 [181.6–198.3] vs control 175.1 [167.9–182.6]). Similar to the intent to treat analysis, there were no differences in anemia, hemoglobin or TFR for mothers or their babies. There was also no evidence of effect modification by maternal nutrition status (low BMI vs. normal BMI) for hematological indicators either prenatally or during the post-partum period (results not shown).

**Table 4:** Hematological indicators from preconception to postpartum by intervention group among women who consumed the supplements for at least 26 weeks (per protocol analysis) ^1^.

Variable	MM	IFA	FA
	(n = 244)	(n = 237)	(n = 260)
**Hemoglobin (g/dL)**			
Prenatal at 1^st^ trimester (<14 wks)	12.14 ± 1.52	12.00 ± 1.22	11.97 ± 1.32
Prenatal at 2^nd^ trimester (14–27.9 wks)	11.25 ± 1.27	11.24 ± 1.34	11.24 ± 1.14
Prenatal at 3^rd^ trimester (≥28 wks)	11.44 ± 1.08	11.41 ± 1.33	11.44± 1.27
Postpartum (3 mo)- Mothers	12.26 ± 1.21	12.37 ± 1.26	12.41 ± 1.38
Postpartum (3 mo)- Children	10.86 ± 1.20	10.59 ± 1.07	10.74 ± 1.27
**Anemia (%)** ^**2**^			
Prenatal at 1^st^ trimester (<14 wks)	20.48	22.85	17.25
Prenatal at 2^nd^ trimester (14–27.9 wks)	36.73	39.61	41.81
Prenatal at 3^rd^ trimester (≥28 wks)	33.67	33.75	33.23
Postpartum (3 mo)- Mothers	40.44	40.45	32.34
Postpartum (3 mo)- Children (< 10.5)	33.89	43.43	36.73
**Plasma Ferritin**^**3**^ **(μg/L)**			
Prenatal	92.79 (86.33, 99.74)	97.79 (90.96, 105.14)	90.92 (85.03, 97.21)
Postpartum (3 mo)	118.15^a^ (109.27, 127.75)	117.78^a^ (108.60, 127.74)	101.52^b^ (93.97, 109.67)
Cord blood	184.27^a^ (176.05, 192.88)	189.93^a^ (181.56, 198.26)	175.09^b^ (167.87, 182.62)
**Plasma transferrin receptor (μg/L)**			
Prenatal	3.52^a^ ± 1.05	3.33^b^ ± 1.04	3.42^a,b^ ± 1.00
Postpartum (3 mo)	4.98 ± 1.31	4.92 ± 1.32	4.83 ± 1.30
Cord blood	8.10 ± 3.23	7.38 ± 3.23	7.77 ± 3.23

^1^Values are mean ± SD for hemoglobin, and transferrin receptor, percentages for anemia and geometric mean and 95% CI for ferritin. Difference among treatment groups were tested using generalized linear regression, adjusting for gestational age, duration of supplements and inflammation. Significant differences (p<0.05) between groups are denoted by different letter superscripts

^2^Non-pregnant women: Hb<12 g/dL; pregnant women: Hb<11 g/dL; children at 3 months: Hb<11 g/dL

^3^ Geometric mean (95% Confidence Interval)

FA- Folic Acid, IFA- Iron and Folic Acid, MM- Multiple Micronutrient

**Fig 2:**
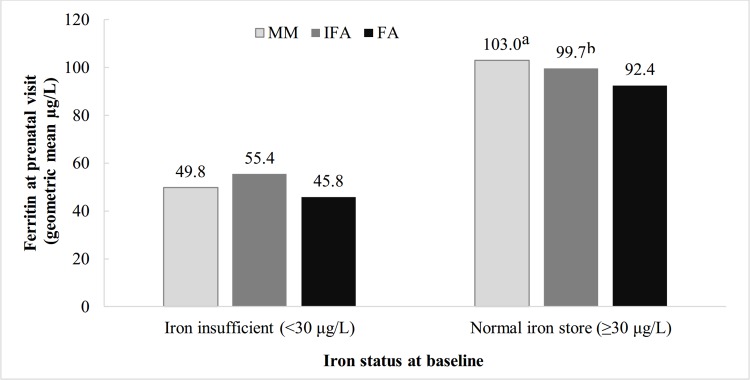
Ferritin at first prenatal visit, by iron status at baseline and treatment group. ^a^ Significant difference (P = 0.003) between MM group and FA group, ^b^ Significant difference (P = 0.03) between IFA group and FA group. Difference among treatment groups were tested using generalized linear regression, adjusting for gestational age, duration of supplements and inflammation. FA- Folic Acid, IFA- Iron and Folic Acid, MM- Multiple Micronutrient.

Mean Hb concentrations during pregnancy were significantly lower among women who were anemic before conception compared to those who were not, but there was no evidence of effect modification by baseline anemia status (S1 Fig). Overall, the prevalence of anemia doubled from 20% at baseline and the first prenatal visit to 40% in the second trimester and then reduced to 33% at the third trimester, with no differences by treatment group (Table 2). We did however find evidence of effect modification by baseline iron status for prenatal ferritin (Fig 2). Plasma ferritin concentrations were 10.6 μg/L (*P* = 0.003) and 7.3 μg/L (*P* = 0.03) higher at the first prenatal visit in the MM and IFA group, respectively, compared to FA among those with normal iron stores (ferritin ≥ 30 μg/L), while no group differences were observed among those with low iron stores (ferritin <30 μg/L). The proportion of women with high ferritin values (>150 μg/L) was 16.5% at the first prenatal visit, and 16% had high hemoglobin concentration (>13 g/dL) [8] during the third trimester of pregnancy. No differences however were observed for high ferritin or high hemoglobin concentrations by treatment group (results now shown).

## Discussion

In this large preconception supplementation trial in Vietnam, we found that weekly supplementation with MM or IFA had no impact on the prevalence of anemia or mean hemoglobin concentrations during pregnancy or postpartum compared to only FA. These results are in contrast to previous evidence primarily from observational studies [20–22,40] and one RCT [41] showing that weekly consumption of iron containing supplements before conception increased hemoglobin concentrations and prevented anemia during pregnancy. However, we demonstrated modest improvements in iron stores during pregnancy among women who received preconception MM or IFA. Furthermore, there were significant increases in plasma ferritin in cord blood and in mothers at 3 months postpartum among women who consumed the MM or IFA supplement for at least 26 weeks, compared to FA. To our knowledge this is the first RCT to study the effect of preconception supplementation of MM, IFA or FA on anemia and iron status during pregnancy and post-partum

Approximately 20% of the women had anemia at baseline. A women’s preconception anemia status was a strong predictor of maternal hemoglobin throughout pregnancy. Despite supplementation both before and during pregnancy, the difference in hemoglobin among anemic and non-anemic women at baseline remained throughout gestation (S1 Fig). This may be due in part to the fact that only 3% of women had iron-deficiency anemia. This contrasts with recent global estimates that suggest 50% of anemia in non-pregnant and pregnant women is amendable to iron supplementation [42]. The differences in the etiology of anemia among different settings could account for the mixed impact of preconception supplementation on anemia in this study compared to others [21,22,41]. The low levels of iron-deficiency anemia at baseline in our study population may explain the lower potential to benefit from supplements compared to other settings where iron deficiency is the primary cause of anemia. This low level of iron deficiency was surprising given the poor dietary quality and high levels of underweight among women in this population [43]. Traditional Vietnamese diets are dominated by starchy staples (mainly rice), are lower in fat and despite increases in protein intake over past two decades only about a third of protein comes from animals sources foods (which have higher bioavailability for several micronutrients) [44]. Prior research in Vietnam has also implicated a low prevalence of anemia (12%) and iron deficiency (14%) despite a high prevalence of other micronutrient deficiencies of public health concern (zinc deficiency (67%) and vitamin B12 deficiency (11.7%) [45]. Likewise, recent studies from Cambodia [46], Malawi [47] and Bangladesh [48,49] have reported a low prevalence of iron deficiency among women.

Other key factors that may contribute to anemia include: other nutrient deficiencies (folate, vitamin B12, and vitamin A), genetic traits (including sickle-cell anemia and thalassaemia), malaria, schistosomiasis, hookworm and trichuris infection, and HIV and some non-communicable diseases [1]. We have previously examined dietary intake in our population using a semi-quantitative food frequency questionnaire at enrollment [43]. Many women were not meeting the estimated average requirement for key micronutrients (percent below estimated average requirement- EAR: 25% for iron, 16% for zinc, 54% for folate, 64% for vitamin B12 and 27% for vitamin A) [43]. Furthermore, there were disparities by income where women in low SES households were at greatest risk for not meeting dietary nutrient requirements. In addition, we have examined factors associated with anemia at baseline in our study population [50] and found that beyond ferritin concentration, being an ethnic minority (-0.24 mg/dL compared with the Kinh ethnic majority), number of children (-0.17) and socioeconomic status (0.09) were directly associated with Hb concentration (*P*<0.05). Retinol Binding Protein (RBP- a marker of vitamin A status) was also directly associated with Hb (0.27 mg/dL) and with ferritin (0.09 mg/dL), whereas hookworm infection was indirectly associated with Hb (-0.11) through RBP and ferritin. However, similar to the low levels of iron deficiency in our population only 4% of women were vitamin A deficient. Thus, based on our existing dietary data we anticipated that other nutrient deficiencies namely B-12 and folic acid may contribute to anemia in this population; however our findings showed no additional benefit of the 15 vitamins and minerals (including iron and folic acid) in the MM supplement compared to IFA or only FA to reduce anemia. Infections and/or inflammation which have been shown to influence biomarkers of iron status [38] did not also appear to be an issue since less than 5% of the cohort had elevated CRP or AGP. Although all women in our study also received the recommended dose of 400 mg albendazole for controlling parasitic infections such as hookworm at baseline, it is possible that the infection was not fully cleared and/or reinfection may have occurred. Finally, we lack data on the prevalence of genetic hemoglobin disorders which may be related to anemia in our population and might have influenced the response to our intervention [46]. Further research is needed to better understand the potential role of genetic traits that may also be affecting iron metabolism in this population.

The impact of supplementation was also examined by baseline anemia and iron status to examine any differences by potential to benefit. Surprisingly the largest difference in plasma ferritin was observed among women who were already iron-sufficient at baseline. This is counterintuitive as we would have expected the more deficient women to respond better to iron supplementation. It is possible, however, that the small proportion of women who remained iron-deficient in this sample were not responsive to micronutrient supplementation, and examining genetic causes or implementing other interventions targeting malabsorption may be needed. It should be noted however that we found no differences by intervention group in the proportion of women with ferritin concentrations over 150 mg/L or elevated hemoglobin (>13g/dL). Thus, while the potential to benefit was low given the relatively iron replete population at baseline, there did not appear to be any harm or risk of iron overload in this population. This is important to establish as countries are beginning to consider preconception supplementation programs and data on micronutrients deficiencies may be limited. The 20% anemia among WRA in this population is at the borderline of WHO recommendations for weekly IFA supplementation. This study provides evidence that both MM and IFA preconception supplementation in iron replete populations provides some benefit of increasing iron stores but does not place women at risk of iron overload.

Our study has several strengths, including the double blinded RCT study design, the large sample size, and the detailed follow-up through pregnancy and postpartum by a well-trained team of local health professionals. The most unique strength is its focus on preconception, and the inclusion of biomarkers of iron status and hematological indices across the continuum from preconception through pregnancy and the first 3 months postpartum. The study however is not without limitations that should be considered when interpreting the results. First, we lack data on other micronutrient biomarkers and genetic information to identify the etiology of anemia in our population. However, we do not expect these characteristics to differ by group due to the randomized design and availability of data showing that the three groups were similar for several socio-demographic characteristics and dietary intakes at baseline. Another concern is the variability in the duration of the intervention as it depended on the time to conception. However, this did not differ by intervention group and we evaluated our results by controlling for it and also conducted a per-protocol analysis that included only women who received the supplements for at least 26 weeks. Finally, it is important to acknowledge that although we found statistically significant differences in plasma ferritin, these differences were small and the biological significance may be limited, especially for women who do not have low ferritin to begin with. There is increasing recognition of the importance of preconception nutrition for fertility, embryogenesis, implantation and placentation, which may have implications for preeclampsia and intra-uterine growth retardation [12]. However it is unclear at what level of nutrient deficiency these processes are impacted. Further research is needed to examine the functional impact of preconception supplementation on neonatal outcomes and other outcomes such as cognitive ability and/or chronic disease may not manifest until later life.

In conclusion, preconception supplementation with MM or IFA in comparison to FA alone resulted in modest increases in prenatal plasma ferritin. In addition, among women who consumed the preconception MM or IFA supplement for ≥ 26 weeks, plasma ferritin concentrations in cord blood and mothers at 3 months postpartum were significantly higher when compared to women who received only FA. Despite high compliance with the interventions both before and during pregnancy, anemia remained prevalent and did not respond to the micronutrient interventions. Further research is needed to characterize the etiology of anemia in this population in order to develop effective interventions for reducing prenatal anemia.

## Supporting Information

S1 Data Dataset. (DTA)

S1 Fig Hemoglobin changes during pregnancy, by anemia status and treatment group. (PDF)

S1 Table CONSORT 2010 Checklist. (DOC)

S1 Text Study Protocol–Method paper. (PDF)

S2 Text Original study protocol. (DOC)
