# Supplementary figures and images for: Impact of Preconception Micronutrient Supplementation on Anemia and Iron Status during Pregnancy and Postpartum: A Randomized Controlled Trial in Rural Vietnam
Source: PLoS One. 2016 Dec 5;11(12):e0167416. doi: 10.1371/journal.pone.0167416 (PMC5137891; doi:10.1371/journal.pone.0167416)

**S2 Figure: Hemoglobin changes during pregnancy, by anemia status and treatment group**

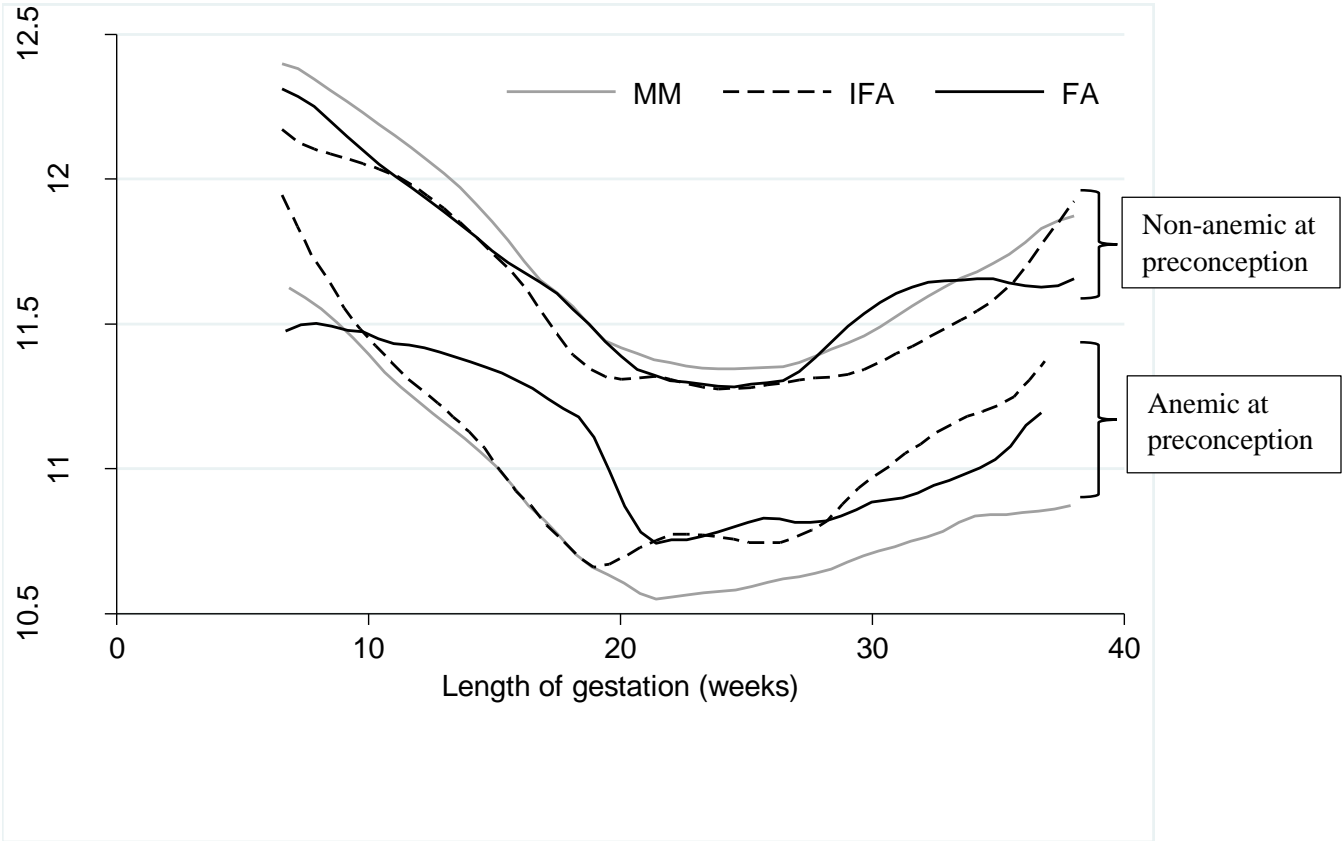

Supplement: S1 Fig — (PDF) [file pone.0167416.s002.pdf]
