# Supplementary material for: Impact of Preconception Micronutrient Supplementation on Anemia and Iron Status during Pregnancy and Postpartum: A Randomized Controlled Trial in Rural Vietnam
Source: PLoS One. 2016 Dec 5;11(12):e0167416. doi: 10.1371/journal.pone.0167416 (PMC5137891; doi:10.1371/journal.pone.0167416)
Supplement: S1 Text — (PDF) [file pone.0167416.s004.pdf]

BMC Public Health

STUDY PROTOCOL Open Access

Rationale, design, methodology and sample characteristics for the Vietnam pre-conceptual micronutrient supplementation trial (PRECONCEPT): a randomized controlled study

Phuong H Nguyen[1,2*], Alyssa E Lowe[3], Reynaldo Martorell[3], Hieu Nguyen[1], Hoa Pham[1], Son Nguyen[1], Kimberly B Harding[4], Lynnette M Neufeld[4], Gregory A Reinhart[5] and Usha Ramakrishnan[3]

Abstract

Background: Low birth weight and maternal anemia remain intractable problems in many developing countries. The adequacy of the current strategy of providing iron-folic acid (IFA) supplements only during pregnancy has been questioned given many women enter pregnancy with poor iron stores, the substantial micronutrient demand by maternal and fetal tissues, and programmatic issues related to timing and coverage of prenatal care. Weekly IFA supplementation for women of reproductive age (WRA) improves iron status and reduces the burden of anemia in the short term, but few studies have evaluated subsequent pregnancy and birth outcomes.

The Preconcept trial aims to determine whether pre-pregnancy weekly IFA or multiple micronutrient (MM) supplementation will improve birth outcomes and maternal and infant iron status compared to the current practice of prenatal IFA supplementation only. This paper provides an overview of study design, methodology and sample characteristics from baseline survey data and key lessons learned.

Methods/design: We have recruited 5011 WRA in a double-blind stratified randomized controlled trial in rural Vietnam and randomly assigned them to receive weekly supplements containing either: 1) 2800 μg folic acid 2) 60 mg iron and 2800 μg folic acid or 3) MM. Women who become pregnant receive daily IFA, and are being followed through pregnancy, delivery, and up to three months post-partum. Study outcomes include birth outcomes and maternal and infant iron status. Data are being collected on household characteristics, maternal diet and mental health, anthropometry, infant feeding practices, morbidity and compliance.

Discussion: The study is timely and responds to the WHO Global Expert Consultation which identified the need to evaluate the long term benefits of weekly IFA and MM supplementation in WRA. Findings will generate new information to help guide policy and programs designed to reduce the burden of anemia in women and children and improve maternal and child health outcomes in resource poor settings.

Trial registration: NCT01665378

Keywords: Anemia, Birth weight, Stratified randomized controlled trial, Supplements, Vietnam, Preconcept

* Correspondence: P.H.Nguyen@cgiar.org
[1]Thai Nguyen University of Medicine and Pharmacy, Thai Nguyen, Vietnam
[2]Poverty, Health and Nutrition Division, International Food Policy Research Institute, Hanoi, Vietnam
Full list of author information is available at the end of the article

BioMed Central

© 2012 Nguyen et al.; licensee BioMed Central Ltd. This is an Open Access article distributed under the terms of the Creative Commons Attribution License (http://creativecommons.org/licenses/by/2.0), which permits unrestricted use, distribution, and reproduction in any medium, provided the original work is properly cited.

Background

Low birth weight (LBW) (birth weight < 2500 g) and anemia remain intractable problems in many developing countries [1-3]. Little progress has been made in reducing the prevalence of LBW despite several decades of program implementation that includes routine daily supplementation of pregnant women with iron folic acid (IFA) through the primary health care system [4,5]. Reasons for low effectiveness include inadequacy of delivery systems, side effects and poor compliance among pregnant women [6]. In many parts of South and South East Asia where LBW is high, ~30% of non-pregnant women and 60% of pregnant women are anemic [3], primarily due to iron deficiency, which has been associated with poor birth outcomes [5,7-11]. Intervening with IFA supplements only during pregnancy however may not be the best approach given the substantial demands for iron by maternal and fetal tissues [12] and the fact that many women in these settings enter pregnancy with suboptimal stores [13]. Weekly IFA supplementation before pregnancy has been considered as an alternative approach to reduce the burden of anemia and improve birth outcomes [14,15]. Weekly IFA supplementation has been shown to improve iron status and reduce the burden of anemia in women of reproductive age (WRA) in the short term, but very few studies have evaluated subsequent pregnancy outcomes. Pre-pregnancy weekly IFA supplementation has been shown to improve iron status and reduce anemia during pregnancy compared to prenatal daily IFA alone in three effectiveness studies [16] conducted in Vietnam [17], Cambodia [18] and the Philippines [19,20]. However no study has assessed the effects of pre-pregnancy IFA supplementation on birth outcomes.

Daily prenatal multiple micronutrient (MM) interventions have also shown promising results. A recent meta-analysis of several intervention trials that compared daily MM to IFA supplementation estimated an overall increase of 60 g in mean birth weight [21]. Other studies reported a reduction of 14% in the incidence of low birth weight [22] and reductions in fetal loss and infant mortality [23]. Providing iron and/or MM pre-conceptually may have even larger effects as they help meet the increased demand for iron and other micronutrients earlier in pregnancy. Results from a recent systematic review suggest pre-conception and peri-conception intake of MM supplements is associated with a reduced risk of preterm birth, LBW and intrauterine growth retardation [24].

Although it is highly plausible that interventions such as weekly IFA or MM supplementation in non-pregnant women may have long term benefits such as improved birth outcomes as well as infant and child growth and development, there is very little empirical evidence from well-designed controlled trials to inform global guidelines or country policy. This double blind stratified randomized controlled trial in Vietnam examines whether pre-pregnancy weekly IFA or MM supplementation improves birth outcomes as well as maternal and infant iron status compared to the current practice of providing only prenatal IFA supplements. This study is a collaborative effort between Emory University, the Micronutrient Initiative, the Mathile Institute for the Advancement of Human Nutrition, and the Thai Nguyen University of Medicine and Pharmacy (TUMP) in Vietnam. This paper will provide an overview of the study design, methodology and sample characteristics from our baseline survey data and note key lessons learned from field experiences during the first year of implementation.

Methods/design

Study design and intervention

PRECONCEPT is a stratified double-blind randomized controlled trial in which eligible women were recruited and randomly assigned to one of three pre-pregnancy groups for weekly supplementation of: 1) 2800 μg folic acid (FA – control), 2) 60 mg iron and 2800 μg folic acid or 3) multiple micronutrients, including 60 mg iron and 2800 μg folic acid. Women who become pregnant receive IFA to be taken daily through delivery, as recommended by WHO [25]. All women are offered deworming at baseline and followed through delivery and three months post-partum. Women who do not get pregnant within 18 months of receiving the intervention will also be followed up and evaluated for markers of iron status.

The micronutrient composition of the supplements provided before and during pregnancy are shown in Table 1. Because FA is universally recommended to WRA for the prevention of neural tube defects, providing a placebo was considered unethical. Therefore, the control supplement includes 2800 μg FA. This dosage is 7 times the minimum recommended daily intake 400 μg/d [15] and has been shown to be safe and as effective as a 400 μg daily dose in improving folic acid and reducing homocysteine levels among WRA [26-28]. The doses of weekly and daily IFA are based on current WHO recommendations for WRA [15]. The composition of the MM supplement was based on the UNIMMAP international multiple micronutrient preparation (UNICEF/WHO/UNU, 1999) used in daily prenatal trials with a few modifications, namely the same amounts of iron and folic acid as the weekly IFA supplement and a higher amount of Vitamin D to be consistent with the recent Institute Of Medicine recommendations [29]. The amounts of vitamins and minerals--other than IFA--are 30-40% greater than the current recommended

Table 1 The composition of iron folic acid and multiple micronutrient supplements

Ingredient	Pre-pregnancy (weekly)			RDA for non-pregnant women
	FA	IFA	MM	
Vitamin A (μg)			800	700
Vitamin D (IU)			600	15
Vitamin E (mg)			10	15
Vitamin C (mg)			70	75
Thiamine (mg)			1.4	1.1
Riboflavin (mg)			1.4	1.1
Niacin (mg)			18	14
Vitamin B_6 (mg)			1.9	1.3
Vitamin B_{12} (μg)			2.6	2.4
Folic acid (μg)	2800	2800	2800	400
Iron (ferrous sulfate) (mg)		60	60	18
Zinc (sulfate) (mg)			15	8
Copper			2 (mg)	900 (μg/d)
Selenium (μg)			65	55
Iodine (μg)			150	150

dietary allowances for WRA. These quantities are safe as the supplements are consumed only once a week and are below the tolerable upper intake level [30].

To ensure quality, we obtained the premix for all formulations from DSM Nutritional Products and had the supplements prepared and packaged under Good Manufacturing Practices standard by a local pharmaceutical factory (*NewCare*) that adhered to quality standards. Samples from each production batch are sent to DSM for quality assurance testing. To ensure adequate blinding of study personnel and participants, we assigned two color-letter schemes for each type of supplement, resulting in 6 unique combinations which are indicated on the blister packages containing the supplements. All supplements were in capsule form, identical in appearance and taste, and coded with lot numbers at the factory that correspond to one of the three treatment arms. Two sealed envelopes containing the code allocations are being stored by two individuals not involved in the study at TUMP and Emory University and will be revealed only after completion of the trial.

Sample size calculations

Based on previous studies that have been done primarily in pregnant women, we chose an effect size of 0.15 SD as worth detecting from the point of view of public health significance. This is equivalent to a difference of 60 g in mean birth weight (assuming a SD of 400 g); 0.27 weeks (~ 2 days) in mean gestational age (assuming a SD of 1.8 weeks); and 2 g/dl in mean hemoglobin concentration (assuming a SD of 1.3 g/dl) between any two groups. Using a one tailed test of comparing means between two groups and a significance level of α=0.05, we estimated that we needed a minimum sample of 550 infants per group (1650 total) to have at least 80% power to detect an effect size of 0.15 or greater [31]. Assuming 10% loss to follow-up and a pregnancy rate (among women who plan to be pregnant) of 35%, we estimated that we should recruit at least 5000 WRA to obtain a sample of 1650 mother-infant pairs.

Study setting

The study is taking place in Vietnam where malnutrition (including chronic energy and micronutrient deficiencies) is common in women and young children. According to the latest national nutrition survey, anemia is present in 28.8% of non-pregnant, 36.5% of pregnant women and 29.2% of children under 5 years of age [32]. The percentage of WRA with low Body Mass Index (BMI< 18.5) was 18% and with BMI ≥ 25 was 8.2% [32]. The rate of LBW is 14% [33], the prevalence of underweight among children under 5 years is 17.5%, stunting 29.3% and wasting 7.1% [32]. The study is being conducted in Thai Nguyen province, the gateway to Vietnam's mountainous northeastern region, located about 80 kilometers northeast of Hanoi. Thai Nguyen is home to 8 ethnic groups and 70% of its 1 million inhabitants live in rural areas. Most residents speak Vietnamese and work as farmers, with the main agricultural products being tea and rice.

Study subjects were identified from 20 communes located in 4 of Thai Nguyen's 9 districts. Communes were selected based on location, availability of health services and population characteristics. The rural communes each have a stable population of approximately 6000 residents of whom 1500 are WRA. A main clinic center, or Commune Health Center (CHC), serves each commune with average of 5 CHC staff and 10–20 village health workers (VHW).

Subject recruitment, randomization to treatment

A list of all WRA was obtained from CHC for each commune. VHW visited the homes of women on the list who were married and not pregnant at the time to inquire about their plans for pregnancy in the upcoming year. Women intending to get pregnant within the next year were invited to participate in the study. The inclusion criteria were: 1) 18–35 years old, 2) currently married, 3) living in one of the 20 communes selected with the intention to stay in the areas for the 24 months following recruitment, 4) plan to have children in the next year and 5) agree to participate with informed consent. The rate of pregnancy before or outside marriage is very low because of strict Vietnamese social norms. Exclusion

criteria included: 1) currently pregnant, 2) regularly consumed IFA or MM supplements in the past 2 months, 3) severe anemia (Hemoglobin [Hb] < 7 g/L; based on testing at enrollment), 4) history of high risk pregnancy and 5) reported chronic hematological diseases, hereditary defects of red blood cells or hemoglobin. Women with severe anemia were referred to the local health clinic for treatment.

As participants were enrolled during baseline data collection they were assigned a unique ID number. Lists of the ID numbers organized by commune were sent to Emory University weekly and were randomized using a SAS generated randomization code to assign women to one of the six supplemental groups. The randomized list was then sent back to TUMP in Vietnam at which point women began their supplement regimen. Group assignment was double-blinded. The dosing regimen is one capsule a week before pregnancy and seven pills a week during pregnancy.

Pre-pregnancy supplement distribution, measurement of compliance and detection of pregnancy

VHWs visit women every two weeks to deliver 2 capsules. The VHW directly observes the consumption of one supplement during her visit, and calls or uses text messages the weeks in between her visits to remind the participants to take the second supplement (cell phone coverage is almost universal in the population). Women are encouraged to consume supplements on an empty stomach at the same time every week to enhance compliance, absorption and minimize side-effects. During each visit, VHWs record the number of supplements consumed and any symptoms or side effects women may have. Compliance is based on a count of empty capsule packets stored in the women's homes. VHWs also ask women whether they have had their menses since the last visit. If women report their last menstrual period to be >5 weeks, they are invited to the CHC for a pregnancy test. Data on last menstrual period are recorded to estimate gestational age. Women who test positive for the pregnancy are scheduled for their prenatal visits at the CHC. Upon confirmation of pregnancy, supplementation with the Preconcept capsules is discontinued and women are given daily IFA instead.

Pregnancy supplementation, monitoring and follow-up during pregnancy

Pregnant women receive prenatal IFA supplements and are advised to take them daily before bed time. VHWs continue to visit pregnant women once every two weeks until delivery to provide supplements, monitor consumption and record morbidity and side effects. All pregnant women are scheduled for 3 prenatal visits: 1) at 6–12 weeks, 2) at 23–28 weeks and 3) at 30–36 weeks at the CHC by trained doctors or nurses. During prenatal visits, doctors record obstetric history, morbidity, workload, depression, vital signs, anthropometric measurements, hemoglobin values, urinalysis results, blood glucose and collect venous blood (for the first and second pregnancy semesters). In addition to standard prenatal care, women with any serious problems are referred to OBGYN department of Thai Nguyen General Hospital. The TUMP study physicians are responsible for following-up on these visits and ensuring appropriate care is received.

Notification of birth and post-partum visit

The VHWs maintain close contact with the pregnant women and their families, especially as the delivery date approaches, and notify the study doctors as soon as a birth occurs. During their third prenatal visit, women receive a delivery card with their VHW's and study doctor's phone numbers. Women are also encouraged to deliver in district hospitals where 90% of deliveries take place. Women are instructed to contact the study staff at the first sign of labor, or ask a family member to do so. The study staff then contacts the hospitals where the woman intends to deliver in order to alert them of the labor and remind them to prepare for data collection at delivery. Trained study doctors or nurses based at district hospitals obtain the delivery data. In special cases, when deliveries take place outside the district hospitals, a study doctor or nurse from the CHC visits the mother in her home or at the delivery site within 7 days after birth to gather delivery data. Women and their newborns are visited at one and three month post-partum to collect postpartum data.

Follow-up with women who do not become pregnant

Women who do not become pregnant will be removed from the study after 18 months of supplement intake. Upon leaving the study, these women will undergo a health assessment and a hemoglobin test on capillary blood using Hemocue®.

Data collection

Study measurements at different time points are listed in Table 2.

Measurement of key outcomes

Birth size: Infant anthropometry is measured as early as possible within seven days after birth and at one and three months postpartum using standard procedures [34,35]. Infant anthropometry measurements include weight, recumbent length and crown-rump length, and the circumferences of head, mid-upper arm and abdomen. All measurements are obtained in duplicate by trained district hospital staff, and a third measurement is

Table 2 Data collection and measurements

Measurements	Pre-pregnancy	Pregnancy			Birth	Post-partum
		6-12 weeks	23-28 weeks	30-36 weeks		
**Maternal**						
Demographics, SES status	√					
OBGYN history	√					
Food frequency questionnaire	√					
Helminthic infections	√					
Mental health	√	√	√	√		√
Morbidity		√	√	√		√
Periodontitis screening		√	√	√		
Workload		√	√	√		
Blood pressure	√	√	√	√		
Anthropometry	√	√	√	√		√
Hematological measures						
Hemoglobin	√	√	√	√		√
Serum ferritin	√	√	√			√
Transferrin receptor	√	√	√			√
Infection indicators (CRP, AGP)	√	√	√			√
Other biological indicators						
Vitamin A	√	√	√			√
Blood Glucose		√	√			
Urine protein		√	√	√		
**Fetal development (ultrasound)**		√	√	√		
**Birth information**					√	
**Child measurement**						
Gestational age					√	
Anthropometry					√	√
Hematological measures						
Hemoglobin						√
Serum ferritin					√	
Transferrin receptor					√	
Infant feeding practices						√
**Supplement information**						
Supplement consumption	√	√	√	√		
Side effects	√	√	√	√		

taken if the first 2 measurements do not fall within a predetermined range.

Gestational age is calculated based on the date of last menstrual period, a reliable method according to previous studies [36]. Ultrasounds are being provided during routine prenatal visits to estimate gestational age and measure fetal growth. Several indicators are being used including gestational sac and yolk sac during first trimester of pregnancy, and biparietal diameter, head circumference, abdominal circumference and femur length during the second or third trimester of pregnancy. In addition, the Farr gestational assessment score is being used at delivery [37,38].

Maternal anemia and iron status: Hb concentrations are measured at baseline, during the three prenatal visits and three months postpartum using Hemocue® [39]. Venous blood samples (5 ml) are collected from women by trained nurses at baseline, during the first and the second prenatal visits and at three months post-partum. Serum ferritin, transferrin receptor (TfR), serum C- reactive protein (CRP) and alpha-1-acid alycoprotein (AGP) are measured using the Sandwich ELISA technique [40].

Infants' anemia and iron status: Infant iron status is measured in cord blood samples (5 ml) obtained at delivery. Plasma ferritin and transferrin receptor are measured by Sandwich ELISA technique [40]. Hb concentration is measured at three months of age from a capillary blood sample using Hemocue® [39].

Other maternal measurement

Demographic data were collected using standardized interviewer-administered questionnaires that include questions about demographic background, ethnicity, marital status, education, occupation, household size and composition.

Socioeconomic status is measured using the asset questionnaire developed by the World Bank for use in developing countries [41] and adapted for local context. The questionnaire includes questions on home and land ownership, housing quality (e.g., house construction materials), access to services (water, electricity, gas, and sanitation services), and household assets including possession of various durable goods, productive assets, and livestock.

Household food insecurity was measured using FANTA/USAID's Household Food Insecurity Access Scale (HFIAS) [42]. HFIAS indicators are validated and provide information about food insecurity at the household level with a specific focus on access-related characteristics of household food insecurity. All the questions use a 30-recall-day period.

Reproductive histories were assessed at baseline by interviewing women about their reproductive history including age at first menses, number of pregnancies, live births, abortions and previous pregnancy complications.

Maternal mental health is examined at baseline, during pregnancy and postpartum. We used the Center for Epidemiologic Studies-Depression Scale (CES-D) [43] at baseline and the Edinburgh Postnatal Depression Scale (EPDS) during pregnancy and postpartum [44].

Maternal morbidity is assessed during the three prenatal visits and two postpartum visits with questions related to fever, cough and cold, diarrhea, constipation and other illness experienced during the past 30 days. In addition, maternal periodontal diseases are assessed by a periodontitis screening questionnaire which includes 10 questions pertaining to signs and symptoms of periodontal diseases and history of periodontal treatment. Previous studies have demonstrated a relationship between maternal periodontal disease and adverse pregnancy outcomes, particularly preterm birth [45-47].

Dietary intake information was collected at baseline using a semi-quantitative food frequency questionnaire (FFQ) developed by the Vietnam National Institute of Nutrition (NIN).

Maternal anthropometric measurements are performed according to standard procedures [35,48]. Maternal height was measured at baseline while all other measurements (weight, mid-upper-arm circumference, triceps and subscapular skinfolds) are taken at baseline, during pregnancy and postpartum. At each time point, all measurements are taken twice. If the first two measurements do not fall within a predetermined range, a third measurement is taken.

Stool samples were collected at baseline. Intestinal helminth infections were examined using the Kato-Katz technique [49]. The main intestinal nematodes observed are *A. Lumbricodes,* hookworm and *T. Trichura.* All women recruited were given the deworming medication Albendazole at baseline.

Maternal workload is assessed during the three prenatal visits by a series of questions asking women to provide details on strenuous activities carried out the day before, the duration of the activity and the frequency of strenuous activity during the last 30 days.

Other information collected in the three prenatal visits includes symptoms of preeclampsia (blood pressure, edema, albumin in urine) and fasting blood glucose in the first and second trimester.

Other child measurements

Infant feeding practice information is collected at one and three months postpartum, using WHO-recommended infant and young child feeding indicators which are widely accepted and used to capture optimal feeding practices in populations [50]. The core breastfeeding indicators will be calculated based on the WHO guidelines [50].

Child morbidity: The incidences of respiratory infection and diarrhea are assessed at one and three months postpartum, concurrent with maternal morbidity evaluations. Any child hospitalization and details of treatment received are also recorded.

Statistical analysis

We plan to assess the impact of the interventions using the "intention to treat" approach for the following outcomes:

1) Maternal iron status at the beginning of pregnancy
2) Birth outcomes (infants' birth weight, birth length and gestational age)
3) Infant's iron status (Hb, ferritin, TfR) at birth and Hb at 3 months of age
4) Maternal iron status at 3 months post-partum

We will also conduct per protocol comparisons that will take into account the compliance and duration of intervention. As a first step, the success of

randomization will be examined by comparing the key baseline characteristics across 3 experimental groups by ANOVA test for continuous variables or Chi-square test for categorical variables. Since the study is still blinded, this comparison is presented in this paper by the 6 colors (2 per treatment group) for key maternal and household characteristics at the time of recruitment (Table 3). To assess the efficacy of the trial, generalized linear models (such as analysis of variance and regression) will be used when the dependent variable is a continuous variable (for example, mean hemoglobin at pregnancy entry) and generalized estimating equation will be used if the dependent variable is a binary variable (for example, prevalence of anemia at pregnancy entry). In all analyses, standard regression diagnostics will be performed to assess model assumptions, including examining distributions, performing any needed transformations, and examining overall fit, residuals and leverage. Statistical analyses will be performed with the SAS statistical package version 9.2 [51]. All statistical tests will be two-tailed and p values < 0.05 will be considered statistically significant.

Table 3 Selected baseline characteristics of the sample by treatment group

	Black	Brown	Green	Orange	Red	Violet	Total
**Maternal characteristics**							
Mean age ±SD	26.2 ± 4.4	26.0 ± 4.6	26.0 ± 4.4	26.4 ±4.7	26.0 ±4.5	26.2 ±4.7	26.2 ± 4.6
Age at first married	21.8 ± 3.3	21.7 ± 3.3	21.8 ± 3.3	21.8 ±3.3	21.8 ±3.3	21.8 ±3.3	21.8 ± 3.3
Minority ethnic (%)	50.2	49.5	49.5	49.2	49.0	49.6	49.5
Education level (%)							
Primary school	8.5	6.5	7.4	8.6	9.1	9.4	8.2
Secondary school	55.3	56.2	57.7	53.1	51.6	54.3	54.7
High school	24.5	25.9	22.4	26.8	27.6	23.2	25.1
College or higher	11.6	11.4	12.6	11.5	11.7	13.1	12.0
Work as farmers	80.2	81.5	78.9	81.6	80.8	80.6	80.6
**Obstetric history**							
Number of children							
0	9.4	11.8	12.6	10.9	10.5	11.9	9.4
1	88.1	84.9	84.7	85.0	85.8	84.9	88.1
≥2	2.5	3.4	2.8	4.1	3.7	3.1	2.5
Last pregnancy experiences							
Prenatal check up	90.6	92.3	88.9	89.2	88.6	90.6	90.0
Taking IFA or MM	75.71	80.2	77.4	78.2	75.6	80.8	78.0
C-section	13.8	14.6	15.1	15.5	16.5	14.5	15.0
LBW (<2500 g)	7.2	7.1	8.1	9.9	8.6	8.7	8.3
Premature delivery	8.0	7.9	10.4	9.7	10.7	9.3	9.3
**Anthropometric measurements**							
Height	152.4 ±5.0	152.8 ±5.3	152.6 ±5.2	152.4 ±5.0	152.4 ±5.2	152.4 ±5.2	152.5 ±5.2
Weight	45.7 ± 5.3	45.7 ± 5.6	45.6 ± 5.5	45.4 ±5.3	45.7 ±5.9	45.5 ±5.7	45.6 ± 5.5
BMI	19.6 ± 2.0	19.6 ± 2.1	19.5 ± 2.0	19.5 ±2.0	19.7 ±2.1	19.6 ±2.1	19.6 ± 2.0
BMI<18.5 (%)	30.5	31.1	33.1	30.3	31.3	31.9	31.4
MUAC	24.5 ± 2.2	24.5 ± 2.3	24.5 ± 2.2	24.5 ±2.2	24.6 ±2.4	24.4 ±2.3	24.5 ± 2.3
Triceps skin fold	15.8 ± 5.1	15.7 ± 5.1	15.9 ± 4.9	15.8 ±5.0	16.2 ±5.1	15.9 ±5.0	15.9 ± 5.0
Subscapular skinfold	14.5 ± 5.0	14.5 ± 5.3	14.5 ± 4.8	14.3 ±5.2	14.8 ±5.3	14.5 ±5.1	14.5 ± 5.1
Calf circumference	31.9 ± 2.1	31.8 ± 2.3	31.8 ± 2.2	31.8 ±2.1	31.9 ±2.3	31.7 ±2.2	31.8 ± 2.2
**Hematological indicators**							
Hemoglobin	13.0 ± 1.4	12.9 ± 1.4	12.9 ± 1.3	12.9 ±1.4	13.0 ±1.4	13.0 ±1.3	12.9 ± 1.4
Anemia (%)	19.7	21.0	19.3	20.4	20.0	18.1	19.7

Ethical considerations

The study design was approved by the Ethical Committee of Institute of Social and Medicine Studies in Vietnam and Emory University's Institutional Review Board, Atlanta, Georgia, USA. The trial was registered in the US Clinical Trials registry (identification number NCT01665378). Written informed consent is obtained from all study participants.

Results - sample characteristics at baseline

Originally, we estimated that recruiting study participants from 16 communes would provide the desired sample size. Careful monitoring of enrollment numbers during the first phase of recruitment (October-December 2011) made it clear that an additional 4 communes would be needed in order to reach the target sample size of >5,000. After a second phase of recruitment (March –May 2012), 5011 participants had been recruited (Figure 1). To date, after 10 months of recruitment, we have had 913 pregnancies and 73 births. The proportion of drop outs before pregnancy is around 10% and lost to follow up during pregnancy is low (results not shown).

Background characteristics of the study samples are presented in Table 3. The mean age of the study subjects was 26.2 ± 4.6 years. Nearly half the women identified themselves as ethnic minorities. More than half completed middle school, 25.1% completed high-school, and 8.2% have less than a primary school education and 12.0% have a college education or higher. The majority of women (80%) work as farmers and the treatment groups do not differ in age, education, or OBGYN history. Nearly a third of women have BMI <18.5 while only 1.7% are categorized as overweight or obese (BMI >25). Nearly 20% of women were anemic at baseline and this differed by commune. There were no difference in the baseline characteristics including measures of maternal nutritional status (based on anthropometry) and anemia by the six treatment groups, suggesting that randomization was most likely effective in ensuring that the treatment groups were similar.

Discussion

To our knowledge, this is one of the first RCTs that evaluate the efficacy of providing weekly IFA or MM before pregnancy in increasing birth weight and duration of gestation as well as maternal and infant iron status. The study is timely in that it responds to the WHO Global Expert Consultation, which identified the need to evaluate the long term benefits of weekly IFA and MM supplementation in WRA [15]. The study is being conducted in a developing country setting in South East/South Asia where anemia and iron deficiency are highly prevalent and will provide valuable scientific evidence that will inform policy and the formulation of appropriate iron or other micronutrient recommendations for WRA. Compared to prenatal supplements, this intervention offer several promising advantages including improved compliance, side effect reduction and affordability.

Although this is an efficacy study, it is successfully using the existing local health system to identify women interested in getting pregnant and to administer a pre-pregnancy intervention, indicating that pre-pregnancy

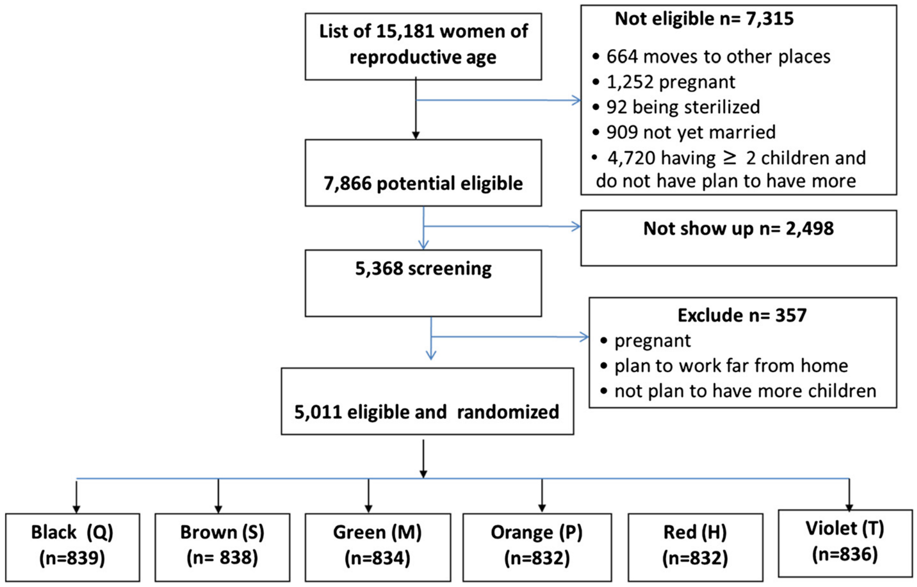

Figure 1 Study recruitment and randomization.

supplementation of WRA is feasible in areas with similar levels of public health infrastructure. Much of the study's initial success can be attributed to the involvement of commune leaders as well as TUMP and its affiliate hospital. Commune leaders and CHC staff were approached early on by the Co-PI in Vietnam, provided with information about the study's purpose and significance, and invited to participate. Commune leaders were recruited to assist with community mobilization, help with the recruitment of village health workers and secure venues for baseline data collection. Continued contact with commune leaders has helped ensure sustained community participation and helped with concerns associated with participants' retention.

The study has also received strong support from influential government authorities. This was accomplished by engaging key leaders and members of health authorities at different levels (provincial, district and commune) at various stages of the study implementation. A unique feature of this study is the extent to which it has been integrated into the existing health network, especially at the commune and village levels. Civic society and local government at the provincial, district and commune levels have also played an important role in providing logistical support and organization. The Vietnam Women's Union mobilized VHWs for supplement distributions and encouraged women to participate in the study. Results will be disseminated to these governmental organizations in order to provide evidence for the revision of policies and programs designed to reduce the burden of anemia and improve maternal and child health outcomes in Vietnam as well as in other countries in the regions with high burden of anemia among WRA including Laos, Cambodia, Bangladesh, India, Nepal [3].

Another important feature of our work has been the focus on building research capacity among medical students and faculty at TUMP. Supervisors were recruited from TUMP faculty and research assistants were recruited from the student body. The local PI along with the Co-investigators serve as important role models and were engaged directly in providing detailed training for all supervisors and research assistants on their specific roles and responsibilities within the larger study objectives. Refresher trainings are provided regularly and personal coaching is provided as needed. Data collection has been standardized and supervision is rigorous and regular. Simple guidelines and procedures for every facet of the study was developed and tailored to the roles and responsibilities of all study staff at all levels: TUMP supervisors, CHC staff and VHWs. Supervision is ongoing at all levels of the study and regular meetings provide the opportunity to trouble shoot problems and respond to issues in a timely manner. The study management board receives updates on the study's progress monthly, outlining study activities in each commune. The monthly work plan (including supplement distribution, supportive supervision, prenatal and post-natal visits, delivery data collection plan, etc.) is specific to each village and organized at the commune level.

Randomization was proven to have been very successful with no significant differences in baseline characteristics among treatment groups. Every effort was made to mobilize key players from all levels in order to ensure the study produces the highest quality data possible. Study results will be disseminated in 3 years. We expect the findings will help generate new information to guide policy and programs designed to reduce the burden of anemia in women and children and to improve maternal and child health outcomes in resource poor settings.

Competing interests
None of the authors had financial or non-financial competing interests.

Authors' contributions
PHN is a co-investigator of the study and contributed to writing proposal, developing the research questions and study design, overseeing data collection, conducting the statistical analysis of data, drafting and revising manuscript. AEL participated in coordinating the study, provided inputs, edited and revised the manuscripts. RM a co-investigator of the study and contributed to developing the research questions and study design, and provided inputs/ comments for the manuscript. HN is a day to-day project coordinator in the field, participated in data collection and provided inputs for manuscripts. HP a day to-day project field director, participated in field supervision, carried out data collection and provided inputs for manuscript. SN participated in field organization and and provided inputs for manuscripts. KBH, LMN and GAR participated in study design and provided comments for the manuscript. UR is the principal investigator of the study and contributed to writing proposal, developing the research questions and study design, overseeing the study, and providing comments/ inputs for manuscript. All authors contributed in the development, review and approval of the final manuscript.

Authors' information
PHN – Phuong H. Nguyen, MD, PhD is a a faculty member of the Department of Scientific Research and International Relationship, Thai Nguyen University of Medicine and Pharmacy, Vietnam, and a research fellow at Poverty, Health and Nutrition Division, International Food Policy Research Institute, Vietnam. tel: +84 0945195395,
E-mail: P.H.Nguyen@cgiar.org
AEL- Alyssa E. Lowe, MPH is a staff of the Hubert Department of Global Health, Rollins School of Public Health, Emory University, Atlanta, GA, USA.
Email: alowe3@emory.edu
RM- Reynaldo Martorell, PhD is a a faculty member of the Hubert Department of Global Health, Rollins School of Public Health, Emory University, Atlanta, GA, USA. Email: rmart77@emory.edu
HN- Hieu Nguyen, MSc is a researcher of the Research Unit, Thai Nguyen University of Medicine and Pharmacy, Vietnam. Email: hieunt1234@gmail.com
HP- Hoa Pham, MD, MPH is a faculty member of the Department of Obstetrics and Gynecology, Thai Nguyen University of Medicine and Pharmacy, Vietnam. Email : hoapham461@yahoo.com
SN- Son Nguyen, MD, PhD is a faculty member of the Department of Pediatrics, Thai Nguyen University of Medicine and Pharmacy, Vietnam.
Email: vansonyk@yahoo.com
KBH- Kimberly B. Harding, MSc is a program officer at The Micronutrient Initiative, Ottawa, Ontario, Canada.
Email: kharding@micronutrient.org
LMN- Lynnette M. Neufeld, PhD is the Director, Technical Srevices at The Micronutrient Initiative, Ottawa, Ontario, Canada.
Email: lneufeld@MICRONUTRIENT.ORG

GAR- Gregory A. Reinhart, PhD is a researcher at The Mathile Institute for the Advancement of Human Nutrition, Dayton, OH, USA.
Email: greinhart@mathileinstitute.org
UR- Usha Ramakrishnan, PhD is the principal investigator of the study and a faculty member of the Hubert Department of Global Health, Rollins School of Public Health, Emory University, Atlanta, GA, USA.
Email: uramakr@emory.edu

Acknowledgement
Funding for this research was provided by the Mathile Institute for the Advancement of Human Nutrition and the Micronutrient Initiative. The authors would like to express special thanks to DSM Nutritional Products from Sight and Life for generous donation of premixes to produce all supplements for the research.

Author details
[1]Thai Nguyen University of Medicine and Pharmacy, Thai Nguyen, Vietnam. [2]Poverty, Health and Nutrition Division, International Food Policy Research Institute, Hanoi, Vietnam. [3]Hubert Department of Global Health, Rollins School of Public Health, Emory University, Atlanta, GA, USA. [4]The Micronutrient Initiative, Ottawa, Ontario, Canada. [5]The Mathile Institute for the Advancement of Human Nutrition, Dayton, OH, USA.

Received: 6 October 2012 Accepted: 11 October 2012
Published: 24 October 2012

References

1. UNICEF/WHO: *Low Birthweight*. New York, NY: Country, regional and global estimates; 2004.
2. UNICEF: *State of the Worlds Children 209*. New York, NY: Maternal and Newborn Health; 2009. 2009.
3. De Benoist B, McLean E, Egli I, Cogswell M: *Worldwide Prevalence of Anaemia 1993-2005: WHO Global Database on Anemia*. Geneva: WHO; 2008.
4. Imdad A, Bhutta ZA: **Routine iron/folate supplementation during pregnancy: effect on maternal anaemia and birth outcomes.** *Paediatr Perinat Epidemiol* 2012, **26**(Supplement 1):168–177.
5. Pena-Rosas JP, Viteri F: **Effects of routine oral iron supplementation with or without folic acid for women during pregnancy.** *Cochrane Database Syst Rev* 2009, **19**(3):CD004736.
6. Ekstrom E: *Supplementation for Nutritional Anemias. Ramakrishnan U, Ed. Nutritional Anemias. Boca Raton, FL*. Boca Raton, FL: CRC Press; 2001.
7. Rasmussen K: **Is there a causal relationship between iron deficiency or iron-deficiency anemia and weight at birth, length of gestation and perinatal mortality?** *J Nutr* 2001, **131**(2S-2):590S–601S. discussion 601S-603S.
8. Ronnenberg AG, Wood RJ, Wang X, Xing H, Chen C, Chen D, Guang W, Huang A, Wang L, Xu X: **Preconception hemoglobin and ferritin concentrations are associated with pregnancy outcome in a prospective cohort of Chinese women.** *J Nutr* 2004, **134**(10):2586–2591.
9. Ross J, Thomas E: *Iron deficiency anemia and maternal mortality. Profile 3 working notes series no. 3*. Washington D.C: Academy for Educational Development; 1996.
10. Zeng L, Dibley MJ, Cheng Y, Dang S, Chang S, Kong L, Yan H: **Impact of micronutrient supplementation during pregnancy on birth weight, duration of gestation, and perinatal mortality in rural western China: double blind cluster randomised controlled trial.** *BMJ* 2008, **337:**a2001.
11. Murphy JF, O'Riordan J, Newcombe RG, Coles EC, Pearson JF: **Relation of haemoglobin levels in first and second trimesters to outcome of pregnancy.** *Lancet* 1986, **1**(8488):992–995.
12. Ramakrishnan U, Semba R: *Iron deficiency and anemia; in Semba RD, Bloem M (eds): nutrition and health in developing countries*. Totawa, NJ: Humana Press; 2008:479–505.
13. Khambalia A, O'Connor DL, Zlotkin S: **Periconceptional iron and folate status is inadequate among married, nulliparous women in rural Bangladesh.** *J Nutr* 2009, **139**(6):1179–1184.
14. Viteri F: **Iron supplementation for the control of iron deficiency in populations at risk.** *Nutr Rev* 1997, **55**(6):195–209.
15. WHO: *Weekly iron-folic acid supplementation (WIFS) in women of reproductive age: its role in promoting optimal maternal and child health. Position statement*. Geneva: World Health Organization; 2009.
16. Cavalli-Sforza T: **Effectiveness of weekly iron-folic acid supplementation to prevent and control anemia among women of reproductive age in three Asian countries: development of the master protocol and implementation plan.** *Nutr Rev* 2005, **63**(12 Pt 2):S77–S80.
17. Berger J, Thanh HT, Cavalli-Sforza T, Smitasiri S, Khan NC, Milani S, Hoa PT, Quang ND, Viteri F: **Community mobilization and social marketing to promote weekly iron-folic acid supplementation in women of reproductive age in Vietnam: impact on anemia and iron status.** *Nutr Rev* 2005, **63**(12 Pt 2):S95–S108.
18. Crape BL, Kenefick E, Cavalli-Sforza T, Busch-Hallen J, Milani S, Kanal K: **Positive impact of a weekly iron-folic acid supplement delivered with social marketing to Cambodian women: compliance, participation, and hemoglobin levels increase with higher socioeconomic status.** *Nutr Rev* 2005, **63**(12 Pt 2):S134–S138.
19. Angeles-Agdeppa I, Paulino LS, Ramos AC, Etorma UM, Cavalli-Sforza T, Milani S: **Government-industry partnership in weekly iron-folic acid supplementation for women of reproductive age in the Philippines: impact on iron status.** *Nutr Rev* 2005, **63**(12 Pt 2):S116–S125.
20. Paulino LS, Angeles-Agdeppa I, Etorma UM, Ramos AC, Cavalli-Sforza T: **Weekly iron-folic acid supplementation to improve iron status and prevent pregnancy anemia in Filipino women of reproductive age: the Philippine experience through government and private partnership.** *Nutr Rev* 2005, **63**(12 Pt 2):S109–S115.
21. Fall CH, Fisher DJ, Osmond C, Margetts BM: **Multiple micronutrient supplementation during pregnancy in low-income countries: a meta-analysis of effects on birth size and length of gestation.** *Food Nutr Bull* 2009, **30**(4 Suppl):S533–S546.
22. Ramakrishnan U, Grant FK, Goldenberg T, Bui V, Imdad A, Bhutta ZA: **Effect of multiple micronutrient supplementation on pregnancy and infant outcomes: a systematic review.** *Paediatr Perinat Epidemiol* 2012, **26**(Supplement 1):153–167.
23. Shankar AH, Jahari AB, Sebayang SK, Aditiawarman, Apriatni M, Harefa B, Muadz H, Soesbandoro SD, Tjiong R, Fachry A, *et al*: **Effect of maternal multiple micronutrient supplementation on fetal loss and infant death in Indonesia: a double-blind cluster-randomised trial.** *Lancet* 2008, **371**(9608):215–227.
24. Ramakrishnan U, Grant F, Goldenberg T, Zongrone A, Martorell R: **Effect of women's nutrition before and during early pregnancy on maternal and infant outcomes: a systematic review.** *Paediatr Perinat Epidemiol* 2012, **26**(Supplement 1):285–301.
25. WHO: *Iron Deficiency Anaemia Assessment, Prevention and Control. A guide for programme managers*. Geneva: World Health Organization; 2001.
26. Adank C, Green TJ, Skeaff CM, Briars B: **Weekly high-dose folic acid supplementation is effective in lowering serum homocysteine concentrations in women.** *Ann Nutr Metab* 2003, **47**(2):55–59.
27. Nguyen P, Grajeda R, Melgar P, Marcinkevage J, Flores R, Martorell R: **Weekly may be as efficacious as daily folic acid supplementation in improving folate status and lowering serum homocysteine concentrations in Guatemalan women.** *J Nutr* 2008, **138**(8):1491–1498.
28. Norsworthy B, Skeaff CM, Adank C, Green TJ: **Effects of once-a-week or daily folic acid supplementation on red blood cell folate concentrations in women.** *Eur J Clin Nutr* 2004, **58**(3):548–554.
29. Institute of Medicine: *Dietary Reference Intakes for Calcium and Vitamin D*; 2010. http:/ www.iom.edu/ Reports/ 2010/ Dietary-Reference-Intakes-for-Calci um-and-Vitamin-D.aspx.
30. Trumbo P, Yates AA, Schlicker S, Poos M: **Dietary reference intakes: vitamin A, vitamin K, arsenic, boron, chromium, copper, iodine, iron, manganese, molybdenum, nickel, silicon, vanadium, and zinc.** *J Am Diet Assoc* 2001, **101**(3):294–301.
31. Cohen J: *Statistical power for the behavioral sciences*. Hillsdale, NJ: Lawrence Erlbaum; 1988.
32. NIN, MOH, UNICEF: *Summary Report General Nutrition Survey 2009–2010*; 2012. http://www.unicef.org/vietnam/resources_18459.html.
33. Khan NC, le Tuyen D, Ngoc TX, Duong PH, Khoi HH: **Reduction in childhood malnutrition in Vietnam from 1990 to 2004.** *Asia Pac J Clin Nutr* 2007, **16**(2):274–278.
34. Lohman T, Roche A, Martorell R: *Anthropometric Standardization Reference Manual*. Champaign, IL: Human Kinetics Publishers; 1988.
35. Cogill B: *Anthropometric Indicators Measurement Guide*. Washington, D.C: Food and Nutrition Technical Assistance Project, Academy for Educational Development; 2003.

36. Neufeld LM, Haas JD, Grajeda R, Martorell R: **Last menstrual period provides the best estimate of gestation length for women in rural Guatemala.** *Paediatr Perinat Epidemiol* 2006, **20**(4):290–298.
37. Farr V, Kerridge DF, Mitchell RG: **The value of some external characteristics in the assessment of gestational age at birth.** *Dev Med Child Neurol* 1966, **8**(6):657–660.
38. Farr V, Mitchell RG, Neligan GA, Parkin JM: **The definition of some external characteristics used in the assessment of gestational age in the newborn infant.** *Dev Med Child Neurol* 1966, **8**(5):507–511.
39. Hemocue: *Hemocue Hb301*; 2012. http://www.hemocue.com/international/Products/Hemoglobin-1155.html.
40. Erhardt JG, Estes JE, Pfeiffer CM, Biesalski HK, Craft NE: **Combined measurement of ferritin, soluble transferrin receptor, retinol binding protein, and C-reactive protein by an inexpensive, sensitive, and simple sandwich enzyme-linked immunosorbent assay technique.** *J Nutr* 2004, **134**(11):3127–3132.
41. Gwatkin DR, Rustein S, Johnsin K, Pande RP, Wagstaff A: *Socio-economic differences in health, nutrition, and population in Bangladesh*. Washington, DC: The World Bank's Health and Population Advisory Service; 2000.
42. Coates J, Swindale A, Bilinsky P: *Household Food Insecurity Access Scale (HFIAS) for Measurement of Household Food Access: Indicator Guide (v. 3)*. Washington, D.C: Food and Nutrition Technical Assistance Project, Academy for Educational Development; 2007.
43. Radloff LS: **The CES-D Scale: a self-report depression scale for research in the general population.** *Applied Psychological Measurement* 1977, **1**:385–401.
44. Cox JL, Holden JM, Sagovsky R: **Detection of postnatal depression. Development of the 10-item edinburgh postnatal depression scale.** *Br J Psychiatry* 1987, **150**:782–786.
45. Offenbacher S, Katz V, Fertik G, Collins J, Boyd D, Maynor G, McKaig R, Beck J: **Periodontal infection as a possible risk factor for preterm low birth weight.** *J Periodontol* 1996, **67**(10 Suppl):1103–1113.
46. Jeffcoat MK, Geurs NC, Reddy MS, Cliver SP, Goldenberg RL, Hauth JC: **Periodontal infection and preterm birth: results of a prospective study.** *J Am Dent Assoc* 2001, **132**(7):875–880.
47. Lopez NJ, Smith PC, Gutierrez J: **Periodontal therapy may reduce the risk of preterm low birth weight in women with periodontal disease: a randomized controlled trial.** *J Periodontol* 2002, **73**(8):911–924.
48. Gibson R: *Principles of Nutritional Assessment*. New York: Oxford University Press; 2005.
49. WHO: *Basic laboratory methods in medical parasitology*. Geneva: World Health Organization; 1991.
50. WHO: *Indicators for assessing infant and young child feeding practices. Part II: Measurement*. Geneva: WHO Press; 2009.
51. SAS: *SAS, the power to know*": SAS Institue Inc; 2002–2004.

doi:10.1186/1471-2458-12-898
Cite this article as: Nguyen *et al.*: Rationale, design, methodology and sample characteristics for the Vietnam pre-conceptual micronutrient supplementation trial (PRECONCEPT): a randomized controlled study. *BMC Public Health* 2012 **12**:898.

Submit your next manuscript to BioMed Central and take full advantage of:

- **Convenient online submission**
- **Thorough peer review**
- **No space constraints or color figure charges**
- **Immediate publication on acceptance**
- **Inclusion in PubMed, CAS, Scopus and Google Scholar**
- **Research which is freely available for redistribution**

Submit your manuscript at
www.biomedcentral.com/submit

BioMed Central
