# Supplementary material for: Impact of Preconception Micronutrient Supplementation on Anemia and Iron Status during Pregnancy and Postpartum: A Randomized Controlled Trial in Rural Vietnam
Source: PLoS One. 2016 Dec 5;11(12):e0167416. doi: 10.1371/journal.pone.0167416 (PMC5137891; doi:10.1371/journal.pone.0167416)
Supplement: S2 Text — (DOC) [file pone.0167416.s005.doc]

**STUDY PROTOCOL**

**Impact of Pre-Pregnancy Micronutrient Supplementation on Maternal and Child Outcomes**

**1. SPECIFIC AIMS AND HYPOTHESES**

The **overall objective** of this study is to evaluate the efficacy of weekly pre-pregnancy iron-folic acid (IFA) and Multiple Micronutrient (MM) supplements on birth outcomes and maternal and infant iron status. The **central hypothesis** is that pre-pregnancy supplementation of weekly IFA or MM followed by prenatal, daily IFA supplements, will improve birth outcomes as well as maternal and infant iron status when compared to prenatal IFA supplementation alone.

The **specific hypotheses** are:

1. Compared to women receiving pre-pregnancy weekly FA, women receiving pre-pregnancy weekly IFA supplements will have:

1. Better iron status during pregnancy, and 1 and 3 months post-partum

b. Infants with larger birth size (weight and length) and longer gestational ages

c. Infants with better iron status at birth, and at 1 and 3 months of age.

2. Compared to women receiving pre-pregnancy weekly IFA, women receiving pre-pregnancy weekly MM supplements will have:

1. Better iron status during pregnancy, and at 1 and 3 months post-partum
2. Infants with larger birth size (weight and length)
3. Infants with better iron status at birth, and at 1 and 3 months of age.

**Figure 1: Conceptual framework relating the impact of providing pre-pregnancy micronutrient supplements in improving maternal and child outcomes**

**Pre-pregnancy iron status and anemia**

Post-partum iron store and anemia

**Maternal outcomes**

**Birth outcomes**

**Infant iron status**

Birth size

Maternal mortality**

Gestational age

Cord blood iron status

Anemia and iron store at 3 mo

Perinatal mortality**

**Pre-pregnancy iron status and anemia**

** 3 groups of weekly pre-pregnancy supplements: folic acid (control), iron and folic acid, and multiple micronutrients.*

*** These outcomes are not being included in the proposed study.*

*Background variables: Dietary intake, dietary supplements, infections, socioeconomic status, education, age, parity, child’s gender*

**2. RESEARCH DESIGN AND METHODS**

**2.1. Study Design**

This study is a **randomized controlled double-blind trial** that tests the hypothesis of whether supplementation with IFA or MM **before** pregnancy combined with IFA supplementation **during** pregnancy is better than supplementation with IFA during pregnancy **alone** in improving the health of pregnant women and their new-born infants. Health outcomes will be assessed at pregnancy, delivery and 1 and 3 months post-partum.

The inclusion and exclusion criteria for recruiting women in the study are as follows:

***Inclusion criteria***

- 18-35 years old
- Currently married
- Currently living in one of the 20 communes and intends to live in the areas for 24 months following recruitment
- Plans to have children in the next year
- Agrees to participate with informed consent

***Exclusion criteria***

- Currently pregnant
- Delivered in the previous six months
- Regularly consumed IFA or MM supplements in the past 2 months
- Severely anemic (Hb < 7 g/L)
- History of high risk pregnancy including abruptio placenta, placenta previa, gestational diabetes, pregnancy induced hypertension, coagulation disorders, thrombocytopenia or chronic vascular, renal or systemic disease or drug use
- Chronic hematological diseases, hereditary defects of red cells or hemoglobin

Eligible women will be recruited and randomly assigned to one of three pre-pregnancy groups for weekly supplementation of:

1. 2800 μg folic acid (FA – control)
2. 60 mg iron and 2800 μg folic acid (IFA) or
3. Multiple micronutrients (MM).

Women who become pregnant will receive daily IFA. The schedule for supplementation is presented in Table 1. All women will be offered routine de-worming and followed through delivery and at one and three months post-partum.

Table 1: Schedule for supplementation

|  | Pre-pregnancy | Pregnancy |
| --- | --- | --- |
| Group 1 | Weekly FA (control) | Daily IFA |
| Group 2 | Weekly IFA | Daily IFA |
| Group 3 | Weekly MM | Daily IFA |

It is unethical to have a placebo group as FA is universally recommended for WRA to prevent neural tube defects. Therefore, the control group will receive 2800 μg FA once a week during the pre-pregnancy period. This dosage is safe and meets the minimum recommended intake of 400 μg/d for WRA (1). The pre-pregnancy and prenatal supplement compositions are shown in Table 2.

**Table 2: The composition of IFA and MM tablets**

| Ingredient | **Before Pregnancy (weekly)** | | | **During Pregnancy (daily)** |
| --- | --- | --- | --- | --- |
|  | **FA** | **IFA** | **MM** | **IFA** |
| Vitamin A (μg) |  |  | 800 |  |
| Vitamin D (IU) |  |  | 600 |  |
| Vitamin E (mg) |  |  | 10 |  |
| Vitamin C (mg) |  |  | 70 |  |
| Thiamine (mg) |  |  | 1.4 |  |
| Riboflavin (mg) |  |  | 1.4 |  |
| Niacin (mg) |  |  | 18 |  |
| Vitamin B_6_ (mg) |  |  | 1.9 |  |
| Vitamin B_12_ (μg) |  |  | 2.6 |  |
| Folic acid (μg)* | 2800 | 2800 | 2800 | 400 |
| Iron (mg)* |  | 60 | 60 | 60 |
| Zinc (mg) |  |  | 15 |  |
| Copper (mg) |  |  | 2 |  |
| Selenium (μg) |  |  | 65 |  |
| Iodine (μg) |  |  | 150 |  |

The doses of weekly and daily iron and folic acid are based on current WHO recommendations for WRA. There is no current recommendation for weekly MM supplements for WRA. Therefore, we propose a supplement that contains:

- The same amounts of iron and folic acid as the weekly, pre-natal IFA supplement
- An amount of vitamin D based on the Food and Nutrition Board’s Recommended Daily Allowances (RDA) (10, 11, 12)
- UNICEF/WHO/UNU recommended amounts of remaining nutrients (UNIMMAP recommendations) (2)

The amount of Vitamin D is based on the most recent RDA, which outdates the UNIMMAP recommendations and recommends a higher daily allowance of Vitamin D (11). Because the supplement will be consumed only once a week, rather than daily, the amounts of vitamins and minerals are 30-40% greater than the most current RDA.

**2.2. Sample size**

We plan to recruit ~ 5500 WRA to obtain a sample of at least 1650 mother-infant pairs.

**2.3. Study setting and population**

The study will take place in Vietnam where malnutrition (including chronic energy and micronutrient deficiencies) is common in women and young children. According to the latest national nutrition survey, nutritional anemia is present in 24% of non-pregnant women, in 32% of pregnant women and in 34% in children under 5 years of age . The rate of LBW is 14% , the prevalence of underweight is 26.6%, stunning is 30.7% and wasting is 7.7% .

The study will be conducted in 20 rural communes in Thainguyen province which is located about 80 kilometers northeast of Hanoi and serves as a gateway to Vietnam’s mountainous northeastern region. With a population of over 1 million people (70% living in rural areas), Thainguyen is home to eight main ethnic groups: the Kinh ethnic groups account for 81% and other minority ethnic groups (Tay, Nung, San Diu, Hmong, San Chay, Chinese and Dao) make up 19 percent of the population.

**3. STUDY TEAM**

This is a collaborative study in which all data collection and laboratory analysis will be done by a developing country institution who will work closely with the investigators at Emory, Micronutrient Inititiave and Mathile Institute. The investigators propose to conduct the study with the **Thainguyen University of Medicine and Pharmacy (TUMP),** Thainguyen. TUMP is a regional medical school which adjoins Thainguyen General Hospital. It is a unique medical center in the mountainous area of North Vietnam with the responsibility of training medical doctors for the regions and to provide medical care for the area. The school has several departments, among them the departments of nutrition and pediatrics, which have been actively participating in conducting research on maternal and child health. Past and ongoing projects include parasitic infections, malnutrition, ways to improve food hygiene and food safety and approaches to ameliorate maternal and child malnutrition and micronutrient deficiencies. Earlier work indicates that the prevalence of anemia and child undernutrition are higher in this part of the country. One survey in 2006 showed that 40% of reproductive age women in this region suffer from nutritional anemia (Duong et al, unpublished).

The resources of TUMP include professional personnel specialized in epidemiologic studies, and technical personnel trained in data collection, management and analysis. The study team in charge of this intervention trial will include:

- Co-PI, Dr. Phuong Nguyen,a trained obstetrician familiar with the study requirements who has worked closely with Drs. Ramakrishnan and Martorell over the past 6 years in her PhD and post-doctoral fellowship at Emory. She played a key role in the development of this proposal while she served as a post-doctoral fellow at Emory. She will be in charge of providing technical support for implementation and quality control.
- The study management board consisting of the Dean of TUMP, the Director of the University Hospital and the Director of Thai Nguyen Health Services Department, who will support the study in obtaining access to districts and communes, permission from government, and who will assist with all other legal issues.
- Field director and co-field director, Dr. Son Nguyen (pediatrician) and Dr. Hoa Pham (OBGYN), who will take charge of all data collection processes, study coordination, technical support, recruiting and training data collectors, and supervising and ensuring the quality of study.
- Study coordinator, Mr. Hieu Nguyen, who will coordinate all the activities of study, including developing study operation manuals, training data collectors, data collection processes, and supervising and ensuring the quality of study.
- 20 TUMP physicians (OBYGN or pediatrician), each in charge of one commune, who will work with the commune doctor and midwife to ensure adequate supplement supply, supervise supplement distributions, follow up with pregnant women and obtain timely data collection
- 20 CHC leaders and 20 CHC midwives, who will manage the work of the CHC health workers. The midwives will provide support for village health workers, and provide antenatal care for pregnant women at the CHCs. They will also assist in following up with pregnant women.
- 227 village health workers belonging to 20 CHCs, who will identify subjects, provide supplements, report pregnancy cases and deliveries.
- 1 data manager and 1 data entry person
- A team of blood collectors who will process, store and ship specimens to Hanoi for analyses.

The example of personnel chart is shown in Figure 2 bellow:


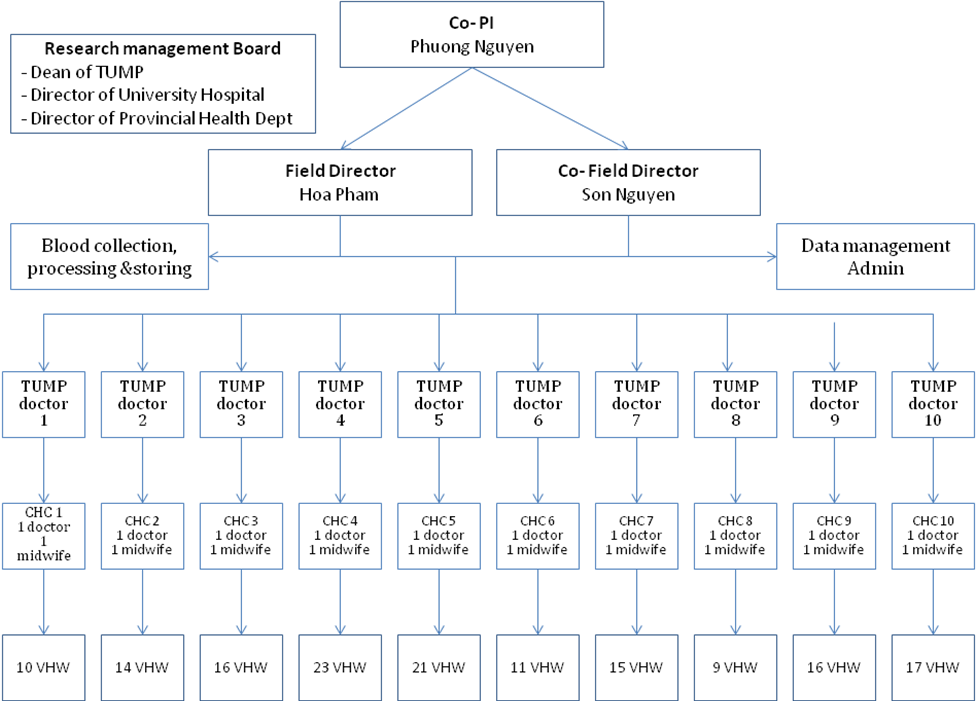


**Figure 2: Field study team**

**4. PROCEDURES FOR STUDY IMPLEMENTATION**

Specific details of the implementation of the trial are provided in the following sections.

***4.1. Identification, enrollment and randomization of study subjects:*** Health information databases maintained by Village Health Workers will help identify women who are married, not pregnant and not currently using a birth control method(s). Field workers will visit the homes of currently married women who are not pregnant to ask whether they intend to become pregnant in the next year. If a women plans to become pregnant in the coming year, she will be invited to participate in the study. Informed consent will be obtained from all subjects following standard procedures. All study protocols will first be approved by the Human Investigations Committees at Emory University and TUMP and explained to the study subjects at recruitment. An assessment of maternal physical and mental health will be conducted. Data on obstetric history, socio-demographic status, anthropometry, dietary intake, iron status and helminth infection will be obtained at baseline. (Helminth infection will be determined through a stool sample.) Venous blood will also be collected at baseline. Women will then be randomized to one of three treatment groups after confirming they meet the study eligibility criteria.

***4.2. Pre-pregnancy supplementation and notification of pregnancy:*** The field workers will visit the women every week to deliver and observe the consumption of the supplement. They will also ask women whether they have had their menses since the last visit. If women report that their last menstrual period (LMP) occurred >5 weeks ago, they will be tested for pregnancy.

**4*.3. Pregnancy supplementation, monitoring and follow-up during pregnancy:*** At the first prenatal visit, venous blood samples will be collected from women in addition to routine measurements that include weight, blood pressure and hemoglobin levels using Hemocue^TM^. Pregnant women will also begin to receive daily supplements of IFA. The field workers will continue to visit the pregnant women twice a month until delivery when they will provide the supplements in a pre-coded container. Women will be instructed to consume one tablet daily preferably on an empty stomach and at the same time each day to minimize side-effects. The number of tablets not consumed and details of side effects will be obtained at each visit. Compliance will be based on pill count. All pregnant women will be scheduled for at least three regular antenatal visits (< 13 weeks, 26-28 and 34-36 weeks of gestation) that will be conducted at the commune clinic by trained nurses. In addition to standard prenatal care, women with any serious problems will be referred to the study physician who will follow-up and ensure that the women receive appropriate care.

***4.4. Notification of birth and measurement:*** The field workers will keep in close contact with the pregnant women and their families especially near the time of delivery and will notify the study nurses as soon as the births occur. Field workers will use cell phone for communication with pregnant women and the study team. A family member will also be identified and designated to notify the study staff immediately after delivery either in person or by telephone. Study nurses will obtain data on the delivery (type of delivery, duration of labor and complications) and birth size measurements (weight, length and head-circumference) within 24 hours of delivery. Details of congenital anomalies and any birth defects will also be obtained. Cord blood samples (3-5 ml) will also be obtained when birth takes place in a hospital setting.

***4.5. Post-partum follow-up:*** Women will be asked to re-consent one month post-partum. A second informed consent will provide women with the opportunity to opt-out of post-partum data collection, in particular infant measurement and infant blood collection. If women agree to participate in post-partum follow up, data on the women’s physical and mental health status, maternal and child anthropometry, data on the child’s health including any congenital anomalies and birth defects as well as infant feeding practices will be collected at 1 and 3 mo post-partum. Infant hemoglobin levels will be determined using a capillary blood sample at 3 months post-partum. Venous blood will be collected from mothers at 3 months post partum.

***4.6 Follow-up with women who do not become pregnant:*** Women who do not become pregnant will be removed from the study after 12 or 18 months of supplement intake. Upon leaving the study, these women will undergo a health (physical and mental) assessment. A venous blood draw and hemoglobin level test using Hemocue will also be performed after 12 or 18 months of supplement intake.

1. **DATA COLLECTION**

Table 3: Details of data collection

|  | **Pre-pregnancy** | | During Intervention(mo) | Pre-natal (weeks of gestation) | | | **Birth** | **Post-partum** (mo) | |
| --- | --- | --- | --- | --- | --- | --- | --- | --- | --- |
|  | Baseline | Supple. | 12 or 18 | < 13 | 24-28 | 32-36 | **Birth** | 1 | 3 |
| Socio-demographic characteristics | **X** |  |  |  |  |  |  |  |  |
| Obstetric History | **X** |  |  |  |  |  |  |  |  |
| Dietary intake | **X** |  |  |  |  |  |  |  |  |
| Assessment of maternal health (physical and mental) | **X** |  | **X** |  |  |  |  | **X** | **X** |
| Maternal Anthropometry | **X** |  |  | **X** | **X** | **X** |  | **X** | **X** |
| Maternal Hb using HemoCue | **X** |  | **X** | **X** | **X** | **X** |  |  | **X** |
| Maternal ferritin, transferin receptor, CRP (venous blood)* | **X** |  | **X** | **X** | **X** |  |  |  | **X** |
| Blood glucose test |  |  |  | **X** | **X** |  |  |  |  |
| Helminth infection (stool sample) | **X** |  |  |  |  |  |  |  |  |
| Supplement consumption |  | **X** | **X** | **X** | **X** | **X** |  |  |  |
| Ultrasound screening |  |  |  | **X** | **X** | **X** |  |  |  |
| Vaginal Swab |  |  |  |  | **X** |  |  |  |  |
| Gestational age and birth outcomes |  |  |  |  |  |  | **X** |  |  |
| Cord blood collection in hospital settings only |  |  |  |  |  |  | **X** |  |  |
| Infant anthropometry |  |  |  |  |  |  | **X** | **X** | **X** |
| Infant iron status (capillary blood sample) |  |  |  |  |  |  |  |  | **X** |
| Infant health and feeding practices |  |  |  |  |  |  |  | **X** | **X** |

**5.1. Measurement of Key Outcomes**

***5.1.1. Birth size***: Infants’ weight, length and head circumference will be measured as early as possible within 24 hours after birth using standard procedures. Infants’ weight will be measured by using a pediatric weighing scale which will be calibrated daily with a known reference weight. Infants will be weighed with a minimum of clothing (e.g., undershirt, underpants or dry diaper). Weights will be recorded to the nearest 0.1 kg. Infants’ length will be measured using a baby-board. Measurements will be read to the nearest 0.1 cm. Head circumference will be measured at the largest occipito-frontal circumference to the nearest 0.1 cm with a non-stretchable tape. Infant mid-arm circumference and mid-calf circumference will be measured. The same data collector will obtain all measurements in duplicate.

***5.1.2. Gestational age*** will be calculated based on the date of last menstrual period. In addition, ultrasounds will be administered at each prenatal visit to confirm gestational age and monitor fetal growth. Hospital records will be used to verify information in a sub-sample of 20% for accuracy. Other details of birth outcomes such as type of delivery, sex, duration of labor and complications, etc, will also be obtained by the trained nurses.

***5.1.3. Mothers’ iron status***

Venous blood samples (5 ml) will be collected from women at: baseline, first prenatal visit and, 3 months post partum. One drop of whole blood will be used to measure Hb concentration using the HEMOCUE B-Hb photometer. The remaining will stay at room temperature for an hour for clotting, following which it will be centrifuged and serum samples will be aliquotted into bar-coded vials. All samples will be stored in an ice box and transported on the same day to TUMP where they will be stored at -20^o^C until analysis.

Hemoglobin levels will also be measured using the Hemocue from a capillary blood sample obtained from pregnant women at their second and third prenatal visits as part of routine prenatal care.

***5.1.4. Infants’ iron status***

Infant iron status will be measured in cord blood samples (5 ml) obtained at delivery and in a capillary blood sample (100 ul) that will be obtained at 3 months of age. The hemoglobin concentration will be measured from a drop of blood using the HEMOCUE B-Hb photometer. The blood samples will be then centrifuged and the plasma will be removed and frozen -20^o^C until analyzing. Plasma ferritin and transferrin receptor concentrations will be assayed using the ELISA method. Hemoglobin will be measured at 3 months of age from a drop of blood using the HEMOCUE B-Hb photometer.

**5.2. Measurement of Potential Confounders-**

*Demographic data* will be collected by using standardized questionnaires that include questions about demographic background, ethnicity, religion, occupation and family situation. These data will be collected by trained field workers who will interview women.

*Socioeconomic status* will be measured using the asset questionnaire developed by the World Bank for use in developing countries.

*Health and reproductive history* will be assessed through guided self-reporting. Women will be asked about their reproductive history such as menstruation, number of pregnancies, live births and abortions, and previous pregnancy complications like: hypertension, convulsions, bleeding, etc. Women will also be asked questions about nervous sickness, depression, psychiatric disorders, and addictions like smoking, drug and alcohol use.

*Maternal pre-pregnant nutritional status*: Pre-pregnant weight and height will be measured at baseline. Weight will be measured using an electronic scale (UNISCALE) that is accurate to 100 g and height will be measured to the nearest 0.1 cm by using a stadiometer.

*Dietary intakes* will be collected at baseline using a single 24-hour dietary recall. The period of recall will be for the day before the interview beginning at 6 am and ending at 6 am on the day of the interview. The administration of this instrument takes approximately 30 minutes. Data entry and conversions from food items to nutrient values (both macro- and micro-nutrients) will be done using a compilation of food composition tables for Vietnamese obtained from Vietnam National Institute of Nutrition.

*Infant feeding practices-* will be assessed at 3 months of age by interviewing the mother. Details of breastfeeding and introduction of complementary foods, supplements, etc will be obtained.

*Stool samples* will be collected at baseline. Women will be given a container during a home visit and will be asked to take a sample of their stool in the morning of the day they are scheduled to go to the clinic and to bring it with them to the clinic. Stool samples will be transported to the parasitological lab at Thainguyen University of Medicine and Pharmacy within an hour. Intestinal helminth infections will be examined using the Kato-Katz technique. The main intestinal nematodes to be observed are *A. Lumbricodes,* hookworm and *T. Trichura.*

Vaginal swabs will be collected during mid-pregnancy to explore the association of *vaginal flora* during pregnancy with birth outcomes.

1. **DATA ENTRY AND MANAGEMENT**

All data forms will be checked for accuracy and completeness by the study supervisors and by the data entry operator at TUMP. Data forms will be sent weekly to the data entry center and will be entered into the project’s computer files using the Epi-Info program. Incorrect entries and missing data will be checked and verified on a systematic basis using range checks for values beyond permissible values and missing values. All data files will be routinely backed up using read-writable CDs and copies will be kept in a locked cabinet at TUMP and Emory University. Only the study team will have access to data.

1. **DATA ANALYSES**

Preliminary descriptive analyses will be done to examine the distribution of the data and check for outliers. Assumptions of normality will be assessed using plots and tests of normality. Non-normal variables will be transformed or categorized as required.

The impact of the interventions on birth outcomes and maternal and infant health will be assessed using the “intention to treat” approach. The main outcomes of interest are:

1) Birth outcomes (infants’ birth weight, birth length and gestational age),

2) Infant’s iron status (Hb, ferritin, TfR) at birth and 3 months of age,

3) Maternal iron status at the beginning of pregnancy and 1 and 3 months post-partum

Effectiveness of randomization will be examined by comparing the key baseline characteristics across 3 experimental groups by ANOVA test for continuous variables or Chi-square test for categorical variables. In the case of significant differences, group differences in the key outcomes will be examined with and without adjusting for these factors. In addition to baseline characteristics such as iron status, sociodemographic status, etc, we will also compare the actual number of doses that women consumed before they get pregnant since the duration of the prepregnancy intervention (measured as time to pregnancy from baseline) and compliance (measured as the % of intended doses consumed) may vary by intervention group.

In addition to adjusting for possible differences, additional analysis that examines the effect of possible dose response will also be conducted. In the case of birth outcomes and maternal and iron status in the post-partum period, we will also take into account the role of compliance during pregnancy and infant feeding practices if there are differences. Generalized linear models (such as analysis of variance and regression) will be used when the dependent variable is a continuous variable (for example, mean hemoglobin at pregnancy entry) and generalized estimating equation will be used if the dependent variable is a binary variable (for example, prevalence of anemia at pregnancy entry). These models account for the correlation among repeated measures in the calculation of both parameter and variance estimates. In both these two models, the intervention group will be the main independent variable (FA, IFA and MM).

In all analyses, standard regression diagnostics will be performed to assess model assumptions, including examining distributions, performing any needed transformations, and examining overall fit, residuals and leverage. Statistical analyses will be performed with the SAS statistical package. All statistical tests will be two-tailed and p values < 0.05 will be considered statistically significant.

**8. QUALITY CONTROL**

Several procedures will be performed to ensure data quality control which is a major concern in these kinds of studies. There will be extensive training of the team of interviewers, and supervision during data collection and data entry. Supervisors will verify all forms for accuracy and completeness on a routine basis before data entry at TUMP. Incorrect or incomplete entries will be corrected during data collection. This will be done using an ongoing interactive system. Monthly reports of all data collection activities will be generated and sent to the study PI at Emory who will also hold monthly meetings by videoconference. Special attention will also be paid to the handling of all biological samples (blood and stool) which will be carefully labeled (type of specimen, identification number and date of collection) using indelible markers and stored in -20 C frost-free freezers after separation and preparation of aliquots on the same day of collection at TUMP. All estimations will be done at TUMP by highly trained and experienced staff. Quality control for the various assays will be monitored by conducting duplicate estimations in a 10% subsample and using external and internal standards of varying concentrations. All data files will be sent regularly to the data manager at Emory who has considerable experience in managing large studies and will be responsible for data cleaning and follow-up with the study investigators and project coordinator in Vietnam. All data analysis will be conducted at Emory in collaboration with all the partners. The key outcome measures will be:

1. Offspring’s size and gestational age at birth
2. Offspring’s iron status at birth and
3. Maternal iron status at the beginning of pregnancy and 3 months post-partum.

The Intention to Treat approach will be used to compare outcomes in the various intervention groups.

**9. STUDY TIMELINE**

The total duration of this project is four years and the time line for the main activities is shown in Table 4 assuming a start date in early 2011.

Activities related to the development of the protocol, pre-testing and finalizing questionnaires, hiring and training of all study staff will be carried out in the first 6-9 months. Screening and recruitment of subjects for the intervention trial will begin in the middle of the first year and will be completed in 6-9 months. The weekly supplementation will begin in the first year of the study as subjects are recruited. Sample size calculations have been made based on an assumption that women will have received at least 3 months of intervention during the prepregnancy period. Women who get pregnant within less than 3 months of prepregnancy supplementation will be followed up however. The total duration of data collection will be about three years and will be completed by the middle of the fourth year. All lab estimations and data entry and cleaning will be carried out in an on-going fashion. Data analysis will be conducted in the last two years and the preparation and submission of manuscripts for publication will be done in the final year. Early findings will be disseminated through short reports and working papers. Important results and conclusions will be disseminated through working papers, journal articles, policy reports and presentations at national, regional and international conferences and meetings.

**Table 4**: Timeline of Key Activities

| Activities | Timeline |
| --- | --- |
| **Pre-Project Activities**  Mathile Institute grant agreement with the MI and transfer of first installment of funds  Study coordination arrangements by the MI with the lead investigator and University | By March 31, 2011  By March 31, 2011 |
| Collaboration arrangements by the University and local collaborator | By April 30, 2011 |
| **Project preparatory activities**:  Submission of Study design for ethics and IRB reviews in US and Vietnam; Meeting of technical team and visit to study site | March - June 30, 2011 |
| Selection of study villages; Procurement of equipment, supplies and supplements; pretesting of tests; and recruitment and training of field personnel | June - July 2011 |
| **Project Activities** |  |
| Screening and recruitment of eligible subjects and baseline data collection | Aug 2011– April 2012 |
| Randomization of treatment and intervention begins immediately after baseline data collection has been completed for eligible subjects. | Aug 2011- April 2014 |
| Measurement of birth outcomes and 3 mo post-partum visits | May 2012 – July 2014 |
| Analysis of biological samples | Oct. 2011 – Oct. 2014 |
| Data collection | Aug 2011- July 2014 |
| Data entry and cleaning | Sept 2011 to Dec 2014 |
